# Advancing Adjuvants for *Mycobacterium tuberculosis* Therapeutics

**DOI:** 10.3389/fimmu.2021.740117

**Published:** 2021-10-25

**Authors:** Ana B. Enriquez, Angelo Izzo, Shannon M. Miller, Erica L. Stewart, Robert N. Mahon, Daniel J. Frank, Jay T. Evans, Jyothi Rengarajan, James A. Triccas

**Affiliations:** ^1^ Emory Vaccine Center, Emory University School of Medicine, Atlanta, GA, United States; ^2^ Yerkes National Primate Research Center, Emory University, Atlanta, GA, United States; ^3^ Tuberculosis Research Program, Centenary Institute, The University of Sydney, Sydney, NSW, Australia; ^4^ Center for Translational Medicine, University of Montana, Missoula, MT, United States; ^5^ Department of Biomedical and Pharmaceutical Sciences, University of Montana, Missoula, MT, United States; ^6^ School of Medical Sciences, Faculty of Medicine and Health, The University of Sydney, Sydney, NSW, Australia; ^7^ Sydney Institute for Infectious Diseases and Charles Perkins Centre, The University of Sydney, Sydney, NSW, Australia; ^8^ Division of AIDS, Columbus Technologies & Services Inc., Contractor to National Institute of Allergy and Infectious Diseases, National Institutes of Health (NIH), Bethesda, MD, United States; ^9^ Division of AIDS, National Institute of Allergy and Infectious Diseases, NIH, Bethesda, MD, United States; ^10^ Department of Medicine, Division of Infectious Diseases, Emory University School of Medicine, Atlanta, GA, United States

**Keywords:** tuberculosis, vaccines, adjuvants, M72, BCG, TLR, Th17, CD4+ T cells

## Abstract

Tuberculosis (TB) remains one of the leading causes of death worldwide due to a single infectious disease agent. BCG, the only licensed vaccine against TB, offers limited protection against pulmonary disease in children and adults. TB vaccine research has recently been reinvigorated by new data suggesting alternative administration of BCG induces protection and a subunit/adjuvant vaccine that provides close to 50% protection. These results demonstrate the need for generating adjuvants in order to develop the next generation of TB vaccines. However, development of TB-targeted adjuvants is lacking. To help meet this need, NIAID convened a workshop in 2020 titled “Advancing Vaccine Adjuvants for *Mycobacterium tuberculosis* Therapeutics”. In this review, we present the four areas identified in the workshop as necessary for advancing TB adjuvants: 1) correlates of protective immunity, 2) targeting specific immune cells, 3) immune evasion mechanisms, and 4) animal models. We will discuss each of these four areas in detail and summarize what is known and what we can advance on in order to help develop more efficacious TB vaccines.

## Introduction

*Mycobacterium tuberculosis* (Mtb) is the causative agent of tuberculosis (TB), an infectious disease that led to the death of 1.4 million individuals in 2019 (1). The current licensed vaccine against TB, a live attenuated strain of *Mycobacterium bovis* known as Bacillus Calmette-Guérin (BCG), is able to provide protection against disseminated forms of disease but is ineffective at providing protection against pulmonary TB in children and adults. Therefore, in order to lessen TB burden worldwide, a more efficacious vaccine and improved vaccine delivery strategies are urgently needed.

The development of more robust TB vaccines has unique requirements that have made progress challenging. Vaccines traditionally provide durable protection by prophylactically inducing neutralizing antibodies that can serve as a first line of defense against pathogens (2). Although numerous pathogens require antibodies for protection, this is less clear in the case of TB. While research on the role of antibodies in TB is ongoing and is of interest due to new data (3), no evidence has yet to suggest that neutralizing antibodies are required for protection against Mtb. Therefore, TB vaccine research has focused on identifying antigens and delivery strategies that maximize the generation of T cell responses. In particular, research has shown that CD4^+^ T cells are a critical component of protective immunity. Mouse studies, however, have demonstrated that T cells are not recruited to the lungs until weeks after infection is established (4). Mtb is able to suppress T cell recruitment and responses by utilizing several immune evasion mechanisms (IEM) to impede antigen-presenting cell (APC) function, thereby dampening adaptive responses. Researchers have attempted to bypass the delayed T cell response by targeting specific APCs such as dendritic cells (DCs), but there is limited evidence that this can preclude the immunosuppressive effects of Mtb upon challenge (5, 6).

Despite these challenges, recent developments have reinvigorated interest in TB vaccine research. A study in humans found that BCG revaccinated adults have increased protection compared to control groups (7). Moreover, while BCG is administered through the intradermal route, a recent study found that administering BCG intravenously can induce robust T cell responses and afford non-human primates (NHPs) protection against Mtb challenge (8, 9). Recent clinical trials of novel vaccine candidates have also yielded promising results. The Phase IIB clinical trial results of the M72/AS01_E_ subunit adjuvanted vaccine demonstrated 49.7% protection against Mtb (10). M72 is a recombinant fusion protein consisting of antigens Mtb32A and Mtb39A and AS01_E_ is an adjuvant that combines monophosphoryl lipid (MPL) with QS-21 (a purified saponin fraction). The AS01 adjuvant system success extends to other infectious disease vaccines as it is used in the FDA approved shingles vaccine “SHINGRIX” and is currently in clinical development for use in malaria (11). These successes set an important threshold for the clinical development of future TB vaccines.

To develop effective antigen-specific T cell responses, host immunity requires non-specific innate cell activation. In the case of a subunit vaccine, priming is accomplished with adjuvants. Modern adjuvants prime host immunity through binding of receptors that recognize pathogen-associated and damage-associated molecular patterns, such as toll like receptors (TLRs), C-type lectin receptors (CLRs), and NOD like receptor (NLRs) on the surface of APCs. Adjuvants are able to activate these receptors and induce downstream signaling pathways such as NFκB signaling, which in turn enables the activation of adaptive immune responses. Given the expense and time-consuming nature of vaccine development, adjuvant compounds and formulations that have been shown to be safe and effective are often repurposed for testing novel vaccine candidates. The majority of current TB vaccine candidates contain adjuvants (**Table 1**). However, a TB-specific adjuvant that is able to induce strong immune responses in the lung but minimize corresponding tissue damage is required. Adjuvanted vaccines delivered directly to the upper or lower respiratory tract may have increased efficacy compared with parenterally administration against respiratory pathogens, such as Mtb. Therefore, development of new adjuvants with defined modes of action will be necessary in order to generate improved vaccination strategies that can provide protective immunity to TB.

**Table 1:** Adjuvants Used in Recent TB Vaccine Candidates.

Adjuvant/Vaccine	Antigen	Adjuvant target	Immune response	Ref
IC31	H56, H1	TLR-9, endocytosis	Th_1_	(12–14)
AS01_E_	M72	TLR-4, lysosomal disruption	B, Th_1_, Th_2_, NK, CTL, DC	(10, 15–20)
GLA-SE	ID93	TLR-4	Th_1_	(21–25)
BCG	Whole cell		Trained innate immunity, Th_1_	(8, 26–31)
MVA85A	Ag85A		Th_1_	(32)
CAF01	H1	Mincle	Th_1_, Th_17_	(33–36)
Advax-CpG	CysVac2	TLR-9	Th_17_	(33, 37, 38)
Lipokel		TLR-2	DC	(39)

Table lists of adjuvant-containing TB vaccine candidates that have recently been tested clinically in humans and pre-clinically in animal models. Known adjuvant targets are presented though other mechanisms of action may also be employed. Abbreviations: AS01, adjuvant system; GLA-SE, Glucopyranosyl Lipid A-stable oil-in-water nano-emulsion; BCG, Bacillus Calmette-Guérin; MVA85A, Modified vaccinia Ankara expressing antigen 85A; CAF, Cationic Adjuvant Formulation; TLR, toll-like receptor, Th, T-helper cell; NK, natural killer cell; CTL, cytotoxic T lymphocyte; DC, dendritic cell.

To address the needs of TB vaccine development and lack of TB-targeted adjuvants, the National Institute of Allergy and Infectious Diseases (NIAID) held a workshop in July 2020 titled “Advancing Vaccine Adjuvants for *Mycobacterium tuberculosis* Therapeutics.” This workshop brought together vaccine and adjuvant developers from industry and academia and researchers in fields beyond TB. It is here that we identified research in four areas that will be essential for development of optimal adjuvants for TB vaccines: 1) correlates of protective immunity, 2) targeting specific immune cells, 3) immune evasion mechanisms, and 4) animal models. In this review article, we discuss these four areas in detail and highlight priority areas which the broader TB research community can address in order to develop efficacious adjuvants and vaccination strategies.

## Correlates of Protective Immunity

### Polarisation of CD4^+^ T Cells

Targeting specific immune cells through passive or active vaccine modalities requires understanding the basic mechanisms of protective immunity to Mtb. Although thorough understanding of protective immunity to Mtb remains incomplete, research has shown that conventional T cells (particularly CD4^+^ T-helper [Th] cells) play a vital role in protection (40). Early mouse studies and clinical data demonstrated that functional Th_1_ CD4^+^ T cells are crucial for protection against Mtb (41–45). Th_1_ immunity is characterised by the secretion of IFN-γ, which aids in microbial clearance by enhancing processes such phagocytosis and secretion of reactive oxygen species in macrophages (46). While these responses have long been a major focus of TB vaccine design, studies have shown that there is a requirement for broader immunity as Th_1_ responses alone are insufficient for protection (47). The MVA85A vaccine trial in humans demonstrated that despite robust induction of ‘multifunctional’ CD4^+^ T cells (producing IFN-γ, TNF and IL-2 cytokines), these immune responses did not translate to additional protection from TB compared to placebo vaccination (32). Some studies even found that excessive Th_1_ polarisation may hinder effective memory responses by producing terminally differentiated T cells that are unable to effectively migrate into the lung parenchyma during Mtb infection (48, 49). More recent studies demonstrated that IL-17 and Th_17_ responses, in addition to Th_1_ responses, are necessary for protective immunity to Mtb (50, 51). Th_17_ cells have the capacity to differentiate into resident memory T cells while IL-17A is a key cytokine required for protection in several pre-clinical models of candidate TB vaccines (33, 34, 37). Th_17_ cells can also secrete additional cytokines that direct recruitment of neutrophils and IFN-γ-producing protective memory CD4^+^ Th_1_ cells during Mtb infection (52, 53). Other studies have also found that CCR6^+^CXCR3^+^ Th_1_/Th_17_ cells responses were present in latently-infected individuals (compared to active infection) and were important for protection in an NHP model of TB (54, 55). But while Th_17_ responses are beneficial, excessive Th_17_ responses are detrimental to the host (56, 57). Therefore, adjuvants for TB vaccines should strive to induce early and balanced Th_1_/Th_17_ as these are more likely to be necessary for protection.

### Other Lymphocytes

While there is evidence that CD8^+^ T cells contribute to protection against Mtb, their role remains debated due to variable findings and inherent differences in human and NHP immune function compared to mice. CD8^+^ T cells can produce cytokines such as TNF, IFN-γ and IL-2 and produce cytolytic granzymes, a feature not shared by CD4^+^ T cells. Of these key cytolytic granzymes is granulysin, which is expressed in human but not murine CD8^+^ T cells, and is capable of direct mycobacterial killing (58). CD8^+^ depletion studies in animal models, however, agree on the role of these T cells in protection. In NHPs, CD8^+^ depletion reduces the protective efficacy of BCG vaccination and infection-induced immunity (59). CD8^+^ knockout mice are also unable to contain Mtb infection, particularly at extended timepoints, suggesting a role for CD8^+^ T cells in protection during chronic stages of infection (60).

Similarly, the function of B cells and humoral immunity in protection against TB has become of particular interest as broader immune parameters are investigated (61). Recent studies indicate the presence of antibodies in humans that are protective against Mtb infection (3, 62). The use of alternative vaccination routes, such as mucosal or intravenous, lead to the generation of pulmonary IgA and antibody-producing lymphoid follicles (iBALT), which has been associated with reduced bacterial burden (8, 26, 33, 37). The generation of inducible lymphoid structures is crucial as they can harbour cells such as CXCR5^+^ CD4^+^ T cells which were found to correlate with a better prognosis of TB disease (63, 64).

While not previously a focus of TB vaccine candidates, CD8^+^ T cells and B cells are often measured as a readout of adjuvant function and may be the contributing factors to vaccine-induced protective immunity (6). The mechanism of the AS01 adjuvant, as determined by pre-clinical animal studies, is thought to be due in part to early IFN-γ production by NK and CD8^+^ T cells (15). In humans, however, a review of TB vaccine candidates tested in the clinic revealed that CD8^+^ T cell responses were relatively poor when compared to CD4^+^ T cells responses (65). Until vaccine efficacy studies in humans become more available, the contributions of B and CD8^+^ T cells cannot be discounted and should be considered in adjuvant development.

### Lung-Localised Immunity

Research has suggested that generating immune responses at the site of infection is crucial for protective immunity. As Mtb is spread *via* the aerosol route, it can use lung-specific cell types to its advantage. The microfold cells found in the nasal associated lymphoid tissue (NALT) and iBALT have been identified as the entry site of the bacterium and granulomas can serve as a niche for the persistence of Mtb (66–68). The need for lung-localized immunity is supported by the fact that local immune cells can respond quickly to infection. The generation of T resident memory (T_RM_) cells is a correlate of protection against Mtb and is an active target in mucosal vaccination strategies (33, 37, 69). Mucosal adoptive transfer of T_RM_ cells from BCG-vaccinated mice into naïve mice revealed that both CD8^+^ and CD4^+^ T_RM_ subsets could afford partial protection against Mtb infection (26). Furthermore, both CD4^+^ and CD8^+^ human T_RM_ cells have been characterised as capable of limiting intracellular Mtb survival *ex vivo* (70). A recent study using human samples also found that the frequency of Mtb-specific T_RM_-like cells that produce IL-17 in the lungs negatively correlated with IL-1ß levels in the blood, suggesting an important role for controlling Mtb growth (71). Additionally, mucosal vaccination could help activate other resident cells, such as γδ-T cells and mucosal-associated invariant T cells (MAIT), that also produce IL-17A (53). While the generation of lung-localized immunity appears to be a significant correlate of protection against Mtb, the challenge remains to validate the safety and efficacy of novel administration methods that generate lung-local immune memory in clinical trials.

### Other Correlates of Protection

Interest in trained immunity as a potential correlate of protection has been driven by the hypothesis that it may be responsible for some of the protective characteristics of BCG. This is supported by studies demonstrating that BCG is able to provide protection against multiple respiratory diseases in addition to TB (27), and the observation that some individuals are capable of early clearance of Mtb without requiring an adaptive immune response (72). While there are multiple vaccine candidates that have been reported to be capable of stimulating systemic innate immune responses, there is emphasis on the generation of lung-resident trained immunity which has been observed after pulmonary and intravenous vaccine administration (6, 28).

Certain cytokines, in addition to those secreted by T cells, also play critical roles in protection. In particular, IL-23 expression was found to be essential for IL-17A-mediated responses against Mtb. Upon aerosol infection with Mtb, naïve murine lungs showed increased expression of IL-17A, which was ablated in the absence of IL-23 (73). IL-23, and to a lesser extent IL-17A and IL-22, is able to lead to CXCL13 production and generation of lymphoid follicles (63). Other studies reported that unvaccinated IL-23-deficient mice are still able to control mycobacterial growth in a fashion similar to wild type animals after exposure to Mtb and BCG as local IFN-γ responses are able to compensate for the loss of IL-17A (73–75). IL-23 plays a compensatory role in the absence of IL-12p70, a key Th_1_ polarising factor, as the addition of IL-23 can also enhance protective immunity against Mtb in the absence of a functional Th_1_ immune response (73, 76). Thus, there is evidence that IL-23 plays a significant role in protective responses which will be important for vaccine-induced immunity.

### Clinical Studies

Insights from clinical trials can help address the knowledge gaps of efficacious TB vaccine design by helping identify correlates of protection. A small number of TB vaccines are currently undergoing clinical trials, including three vaccines that incorporate adjuvants. The most advanced of these TB vaccines is the M72/AS01_E_ construct. The MPL components of the adjuvant, a derivative of the lipopolysaccharide from *Salmonella minnesota*, is commonly used in adjuvant formulations due to its ability to engage TLR4, activate NF-kB, and induce pro-inflammatory cytokines (16, 17). QS-21 can cause lysosomal disruption and consequent Syk activation, as well as NLRP3 inflammasome activation, which is thought to enhance cross-presentation with CD8^+^ T cells and promote inflammatory cytokine production (18, 19). Preclinical studies suggest that the Th_1_ polarising effects of AS01 are the result of MPL and QS-21 synergy. At early timepoints post-vaccination, it was observed that subcapsular sinus macrophages (SSM) in the draining lymph nodes promoted early IFN-γ production by resident NK cells and CD8^+^ T cells in a process mediated by IL-18 (15). In humans, the peripheral immune response has been examined by analysing blood RNA expression and antigen-specific PBMC profiles during a two dose M72/AS01_E_ regimen (77). PBMC restimulation showed that the vaccine induced CD4^+^ T cells and multifunctional T cells after stimulation, though IL-17A was not detected. RNA analysis identified the upregulation of blood transcription modules associated with IFN signalling, innate activation including TLR and inflammatory signalling, as well as modules related to various chemotactic and cell adhesion processes.

The cationic peptide adjuvant IC31 is a component of two TB vaccine candidates undergoing clinical trials, H4:IC31 and H56:IC31 (7, 12). In a Phase IIB trial, which tested the efficacy of H4:1C31 and BCG revaccination, H4:IC31 did not demonstrate significant protection (30.5% efficacy) against either initial or sustained Mtb infection (7). However, BCG revaccination led to a 45.4% reduction in sustained infection (7). IC31 is made up of the antimicrobial peptide KLKL_5_KLK (KLK) combined with ODN1a, a TLR9 binding single stranded oligodeoxynucleotide (ODN) that activates the MyD88 pathway (13). The cationic peptide component is also an immunostimulant, hypothesised to enhance intracellular TLR access of ODN1a *via* stimulating endocytosis (14). H56:IC31 induces antigen-specific IgG responses and Th_1_ cytokine expressing CD4^+^ T cells (12). Low-dose vaccine administration induced more polyfunctional memory T cells than high dose vaccination, an observation in line with pre-clinical studies that identified lower antigen dose as conducive to more protective immune responses (78).

Comparative analysis of human immune responses to six TB vaccine candidates observed that a shared feature of the systemic immune responses induced by TB vaccine candidates was the enhanced production of IFN-γ expressing CD4^+^ T cells, with M72/AS01_E_ inducing the greatest response (65). Furthermore, little to no IL-17A expression was induced by the candidate vaccines. This study did not include analysis of CAF01, a cationic liposomal formulation consisting of DDA liposomes and the Mincle agonist trehalose-6,6-dibehenate (TDB), which is known to initiate a Th_17_ response when administered parenterally in mice (79). Vaccination of humans with H1-CAF01, however, induced strong antigen-specific Th_1_ responses while IL-17 responses were low and were not significantly increased (35). This may reflect a requirement to examine mucosal immune responses in humans to better reflect vaccine immunogenicity, as was performed with the CTH522/CAF01 chlamydia vaccine candidate (80). Overall, there was a lack of diversity in T cell responses generated by the different TB vaccine candidates, reinforcing the argument for a requirement to develop and test more novel adjuvants that induce distinct immune responses.

### Adjuvating Strategies for Inducing CD4^+^ T Cells

Differential receptor activation, with adjuvants, have been demonstrated to be important for dictating specific T cell responses. Th_1_ differentiation *via* TLR activation and downstream IL-12 secretion is believed to play a primary role in the protective efficacy of TLR4-targeting adjuvants such as AS01 (15). Similarly, the major driver of immunogenicity of the vaccine candidate ID93 + GLA-SE vaccine is the synthetic TLR4 agonist, GLA; its delivery in a squalene emulsion is also necessary for adjuvanticity (21). The Th_1_ polarising properties of GLA-SE is MyD88- and TRIF- dependent, and type I and II IFN expression is also critical for the adjuvant mode of action (21, 22). Furthermore, IL-18 and Caspase1/11 expression is required for T cell activation by GLA-SE, but not the NLRP3 inflammasome (23). In humans, ID93 + GLA-SE vaccination results in the generation of multi-cytokine producing T cells (TNF, IFN-γ and IL-2), with little IL-17A detected, and IgG1 and IgG3 antibody production (24, 25). Thus, both MPL adjuvant (in AS01) and GLA (in a squalene emulsion) have demonstrated the ability to enhance T cell immunity to Mtb in combination with different antigens, mainly through TLR4-mediated Th_1_ polarisation.

Some adjuvants are innately capable of stimulating Th_17_ polarisation, often by activating non-TLR pattern recognition receptors such as Mincle (81). It is known that some TLR4 and TLR7/8 agonists can induce IL-23 expression, and Mincle-activating adjuvants such as TDB, used in the cationic liposome formulation CAF01, can also shift the balance towards IL-17A producing T cells (36). It is thought that TDB, a derivative of the mycobacterial cord factor (TDM), is the major contributor to the Th_17_ polarisation in this vaccine formulation. Mincle activation, as well as MyD88 and the inflammasome component ASC, have all been identified as a requisite for the Th_17_ generating characteristics of TDB (79, 82, 83). Thus, there are novel adjuvant strategies focused on generating synthetic aryl-trehalose derivatives that afford the best Th_1_ and Th_17_ polarisation (84, 85). Similarly, it has been observed that adjuvants such as cyclic dinucleotides and chitosan that activate the cGAS-STING (cyclic GMP-AMP synthase-stimulator of interferon genes) pathway also stimulate Th_1_ and Th_17_ responses (86–88). As mentioned above, mucosal delivery is another effective strategy for the generation of Th_17_ responses, with many vaccines displaying enhanced Th_17_ polarisation upon mucosal vaccination that was not observed with parenteral administration. Evaluation of these new Th_17_-inducing adjuvants alone and in combination with other established adjuvant systems in relevant animal models is a critical next step in the advancement of improved vaccination strategies for Mtb.

In summary, data supports the development of adjuvants and vaccines that elicit both local (tissue resident) and systemic antigen-specific Th_1_ and Th_17_ cells as these responses have been demonstrated to be critical for protection. However, research should continue to elucidate correlates of protection to identify new pathways that can be targeted by adjuvants to induce protective immunity against Mtb.

## Targeting Specific Immune Cells

Active or passive targeting of specific immune cells through vaccination is an important step in the development of a safe and effective vaccine for TB. Mtb is primarily transmitted *via* inhalation and establishes infection in the lung through phagocytic uptake of the bacilli by tissue-resident macrophages. The initial recognition and immune activation by innate immune subsets sets the stage for either clearance or persistent containment within a granuloma or active disease through suppression of the immune system (89). Cells involved in lung-specific innate and adaptive immune responses are important frontline targets for Mtb vaccination approaches. The majority of Mtb vaccines are delivered *via* the intramuscular (such as M72/AS01_E_) or intradermal (BCG) route, creating additional challenges in the recruitment of tissue resident memory cells to the site of initial infection. Targeting vaccines to specific cell types could help to overcome some of the shortfalls of current vaccine approaches while improving both safety and efficacy. Several groups have worked to overcome these challenges through targeting specific immune cells *via* passive (adjuvants, delivery systems) or active (mucosal vaccination, prime-pull, receptor targeting) immunization strategies with great success in pre-clinical and early clinical investigations. Coordination of the innate and adaptive immune response is important for resolution of Mtb infection and targeting of specific immune cells that orchestrate this response at the site of infection is critical in the development of an effective vaccine.

The context in which antigens are presented to the immune system controls the immunological outcome of antigenic exposure (90). The innate immune system uses pathogen recognition receptors (PRRs) to decode the nature of the antigen (*e.g.*, viral, bacterial, fungal) and to translate this into an appropriate adaptive immune response. Central to the idea of cell-targeting is activation of the innate immune response by pathogen associated molecular patterns (PAMPs) and the specific cellular targets they encounter are crucial for early control of infection and for the subsequent development of protective long-term adaptive immunity. The use of passive and active cell targeting strategies opens the door towards the rational design of vaccines for Mtb that could lead to more durable and protective mediated immune responses.

### Cell Targeting *via* PRR Expression and Adjuvant Use

To drive the desired adaptive immune response, the innate immune response must be properly activated to provide the correct signals for differentiation of antigen-specific T and B cells. Different adjuvants activate specific cell types as a result of differential PRR expression. The directed, rational use of adjuvants is therefore one way in which specific innate immune cells can be targeted in the context of Mtb vaccination. Many adjuvants being explored pre-clinically or in early clinical trials for Mtb vaccines target and activate DCs. In the context of Mtb, several studies have shown that directly targeting DCs can lead to protective immune responses during infection (5, 91–93). As previously discussed, MPL of AS01 activates TLR4 (15). DCs express high levels of TLR4 and are highly responsive to MPL, driving downstream antigen-specific T cell differentiation (11, 20). Both myeloid DCs (mDCs) from blood and monocyte-derived DCs (moDCs) express TLR4 and are able to upregulate co-stimulatory markers and secrete Th_1_ polarising cytokines (in particular IL-12p70) after TLR4 stimulation (94). DCs also express high levels of TLR2 (94). Adjuvants that engage TLR2 on DCs have the potential to augment early production of pro-inflammatory cytokines such as IL-6, IL-1β, TNF-α and IL-12 that are necessary for generating balanced Mtb-specific Th_1_/Th_17_ responses (95, 96). This is demonstrated by studies using Lipokel, an adjuvant which stimulates TLR2 through binding of the ligand Pam2Cys. A protein-Lipokel vaccine conjugate was able to reduce Mtb CFU in the lungs of mice and increase the frequency of DCs in lymph nodes following vaccination (39). PRR-targeting adjuvants, including some TLR agonists, can also preferentially target and activate monocytes and macrophages in addition to DCs. Monocytes and macrophages are specialized phagocytic cells that are capable of interacting with and activating antigen-specific T cells (97). TLR2, TLR4, and TLR8 are highly expressed on monocytes (98). In addition to activating DCs, AS01_E_ was also able to activate SSM innate immune cells (15). The ability of AS01_E_ to target both DCs and monocytes/macrophages concurrently may contribute to success as an adjuvant in vaccine strategies for a variety of pathogens, including Mtb. Additionally, non-TLR PRR agonists, such as Mincle or Dectin-1 ligands, preferentially target monocytes and macrophages due to their high Mincle expression (99). CAF01 has demonstrated efficacy as an Mtb vaccine adjuvant in pre-clinical mouse models and induces a strong Th_17_ polarised immune response in mice (36). Interestingly, when used as an adjuvant in human clinical trials, CAF01 elicited an antigen-specific Th_1_ response instead of a Th_17_ response (35). TDB, the main component of CAF01 believed to be responsible for Th17 polarisation, does not appear to be a particularly potent human Mincle agonist relative to its potency in mice, therefore it is possible that more potent human Mincle agonists may be required to elicit antigen-specific Th_17_ immunity (84). Given the complex nature of various PRRs and their cellular targets in natural Mtb infection and resolution, it is likely that a combination adjuvant and delivery system approach (similar to AS01) and innovative antigen design may be necessary to improve vaccine efficacy. Promising approaches include combining CLR/TLR adjuvants (presented at this workshop) and other novel antigen and adjuvant combination approaches, such as CysVac2/Advax^CpG^, have shown promise in pre-clinical models (38).

### Cell Targeting *via* Mucosal Administration

Most vaccines are injected into muscle although BCG is given as an intradermal injection. However, mucosal immune subsets that have the ability to quickly transport antigen to stimulate an immune response may be important targets for mucosal Mtb vaccines (66, 100). T_RM_ can be induced by mucosal vaccine administration (9, 26, 101, 102). Following mucosal vaccine administration or natural infection, lung resident T cells acquire a polyfunctional phenotype and are more likely to reside in the airway lumen and lungs, where they can rapidly respond to Mtb (40, 103–106). These lung-resident T cells are not typically generated as a result of parenteral vaccination, and it seems likely that mucosal or lung-resident innate cells are crucial for the development and differentiation of Mtb-specific lung-resident T cells. These important innate immune effector cells can be uniquely targeted *via* intranasal and/or intrapulmonary vaccination. Several groups have explored a “prime-pull” strategy which takes advantage of both parenteral vaccine (prime) and mucosal vaccine boost (pull) to direct antigen-specific T_RM_ to the lungs (107, 108).

There are a small number of mucosal vaccines currently in clinical use and all but one (FluMist, intranasal) are delivered orally (109, 110). However, there are many preclinical adjuvants that have been tested for either intranasal or intrapulmonary administration, with a particular bias towards intranasal administration due to improved safety profile compared to intrapulmonary administration and lower requirement for specialised equipment (111, 112). Particulate adjuvants such as carbohydrate or PLGA have also been used extensively for mucosal vaccination, often chosen for their mucoadhesive properties (113). Naturally-derived carbohydrate particles such as delta inulin (Advax), chitosan, and *Bacillus subtilis* spores have all been utilised as mucosal adjuvants in TB vaccine candidates, all producing a Th_1_/Th_17_ phenotype alongside increased pulmonary IgA (37, 114–116). CAF01 has been tested intranasally and has also been spray-dried for intrapulmonary administration (107). Th_17_ immunity is often achieved *via* mode of administration, as it has been observed across multiple vaccine platforms that mucosal vaccination, particularly intranasal, favours Th_17_ differentiation (117, 118). Many adjuvants have the capacity to be Th_1_ or Th_2_ polarising when administered parenterally but promote Th_17_ differentiation when delivered mucosally (37, 119, 120). Some TB vaccines, such as BCG or the CysVac2/Advax candidate, have been observed to be more protective after intranasal or intrapulmonary administration, a quality attributed to local IL-17 production and the establishment of T_RM_ (37, 119). This was observed when the clinical candidate vaccine ID93 + GLA-SE was administered through a different route; parenteral administration induces a Th_1_ responses but intranasal administration induces a Th_17_ responses (121).

### Receptor-Mediated Active Cell Targeting

DCs and macrophages are a primary focus of vaccine design due to their role as APCs and critical function in orchestrating long-term cell-mediated immunity. The tissue heterogeneity in both DC and macrophage populations creates challenges for both passive and active targeting of the various systemic and tissue-specific APC subsets. Active targeting to specific APC subsets through endocytic receptors is a promising approach to improve vaccine efficacy and reduce unintended effects (122). Such receptors include DEC-205, Clec9A, Clec12A, and DC-SIGN, among others (122–126). Early work demonstrating active targeting by DEC-205, a cell surface receptor involved in the uptake of dying cells and cross-presentation of antigens, led to the evaluation of an anti-DEC-205-Ag85B vaccine conjugate for Mtb (125). While strong Ag85B-specific humoral immunity was noted following vaccination, cell-mediated immunity was lacking without BCG priming of vaccinated mice and the vaccine failed to improve protection from Mtb challenge (125). A similar approach was used to target DC-SIGN; anti-DC-SIGN antibodies conjugated to Ag85B were used to vaccinate an hSIGN transgenic mouse (human DC-SIGN under control of the murine CD11c promotor) in combination with various adjuvants. This innovative vaccine design induced strong antigen-specific CD4^+^ T cell responses. However, similar to the DEC-205 approach, enhanced protection from Mtb challenge was not achieved (126). Improved humoral and/or cell-mediated immunity to Ag85B was measured using both endocytic receptor APC targeting strategies demonstrating proof-of-concept for improving immunity using this approach. Additional research efforts are required to identify the appropriate combination of antigen(s), targeting mechanisms, and adjuvants to drive durable immunity and protection in Mtb animal challenge models.

### Targeting Cell Specific Responses Through Trained Immunity

In the previous section, we briefly discussed the potential of trained immunity to serve as a correlate of protection. The contribution of trained immunity to vaccine-mediated protection against Mtb is currently being investigated (127, 128). PRR agonists, among other factors, can drive functional and epigenetic reprogramming in innate immune cells in order to increase Th-polarising cytokines and phagocytic and cytotoxic killing capacity. BCG, when administered intravenously, provides both short and long-term protection from subsequent Mtb challenge in NHPs (8, 28–31). A recently discovered mechanism of protection by intravenous BCG is the generation of trained immunity in hematopoietic stem cells (HSCs). Following intravenous administration, BCG is able to enter the bone marrow (BM) where it can be detected for up to 7 months after vaccination in mice (which is not observed in subcutaneous vaccination) (28). Once in the BM, BCG promotes myelopoiesis and induces trained monocytes and macrophages (28). These BM monocytes have a particular transcriptional and epigenetic program, allowing them to differentiate into trained lung macrophages and mount a rapid protective response against Mtb challenge (28). A similar trained immunity effect on HSCs was found through the use of β-glucan, a Dectin-1 agonist, administered *via* intraperitoneal injection (129). Subsequent work showed that mice with β-glucan-induced trained HSC-derived monocytes and macrophages were significantly better protected from Mtb challenge (130). Therefore, targeting trained immunity through vaccination will require unique adjuvants capable of eliciting trained lung resident macrophages either locally or through HSC-derived cells. Data suggests this may be accomplished through the use of live-attenuated vaccines with endogenous adjuvants (*e.g*., BCG) or exogenous adjuvants in combination with a subunit vaccine (*e.g*., β-glucan or other CLR agonist). Additionally, it may be possible to elicit trained immunity specifically in lung macrophages and DCs *via* mucosal vaccination routes (131).

In summary, using adjuvants that activate PRR and other key receptors on innate immune cells and are administered through the mucosal route hold the most promise in inducing adaptive immune responses. Additionally, being able to generate adjuvants that activate processes such as trained immunity could be beneficial for inducing protective immunity to TB.

## Immune Evasion Mechanisms

An important reason for the poor efficacy of natural immunity to Mtb or BCG vaccine-induced immunity is immune evasion mechanisms that lead to ineffective crosstalk between innate and adaptive immunity (95, 132). By identifying critical Mtb factors that prevent optimal innate immune responses and elucidating the molecular basis for how host pathways are subverted by Mtb, we can engineer vaccines that target specific pathogen and host pathways to improve the immunogenicity and efficacy.

Mtb has evolved multiple strategies to evade innate immunity and impede T cell responses. By impairing DC functions (i.e., co-stimulation, cytokine production and antigen presentation) during early stages of infection in the lung, Mtb induces delayed and suboptimal antigen-specific T cell responses that fail to eradicate infection or provide lasting protection (4, 133–136). Vaccine strategies need to augment the induction of additional Mtb-specific T cell subsets, such as Th_17_ cells, that will work in concert with Th_1_ responses to enhance protective immunity (137). Mtb also inhibits macrophage microbicidal functions and dampens production of early proinflammatory cytokines and chemokines critical for shaping the nature and magnitude of T cell subsets that home to the site of infection (95, 138–140). Moreover, emerging evidence that myeloid-derived suppressor cells (MDSC) (141) and/or neutrophils (142) suppress T cell responses to Mtb infection suggests that targeting these cell types may improve vaccine-induced immunity.

Several Mtb genes have been implicated in evading DC and macrophage responses and, when deleted in BCG or Mtb, show enhanced vaccine-induced protection against TB in animal models. Thus, removing immune evasion genes shared by Mtb and BCG is an important approach for improving live attenuated vaccination strategies. The following studies are examples of applying knowledge gained from mechanistic studies of Mtb immune evasion genes and the host pathways that they modulate towards designing better vaccines for TB.

Inhibition of apoptosis as an immune evasion strategy is exemplified by the *nuoG* gene in Mtb, which encodes a subunit of NADH dehydrogenase and inhibits macrophage apoptosis (143, 144). Infection of mice with MtbΔ*nuoG* led to an increase in apoptosis along with earlier activation of T cells compared to WT, suggesting that *nuoG* dampens the ability of innate cells to initiate T cell responses (145). Moreover, deleting *nuoG* in BCGΔ*ureC*::*hly*, a recombinant vaccine strain that is more protective than BCG in animal models, led to increased apoptosis following vaccination and significantly lowered bacterial burdens in Mtb-challenged mice (146). Deletion of Mtb *sigH*, which regulates multiple stress-induced proteins in Mtb, also led to increased apoptosis and chemokine responses in infected macrophages relative to wild type (147, 148). Mucosal vaccination with Mtb*ΔsigH* in NHPs resulted in increased survival and reduced lung pathology following challenge compared to BCG, with higher central and effector memory T cells in the lung (9).

Cell surface proteins are well positioned to modulate innate immune functions. The Mtb serine protease Hip1 is present in the cell wall of Mtb and dampens macrophage and DC functions *via* proteolytically cleaving its substrate, Mtb GroEL2 (138, 149). Hip1 inhibits TLR2 and inflammasome-dependent macrophage proinflammatory responses, impairs CD40-mediated costimulatory responses in DCs, and restricts Th_17_ polarisation during infection (91, 136, 138). Both an Mtb *hip1* mutant and BCGΔ*hip1* strain augmented CD40 expression on DCs, enhanced macrophage and DC functions and led to higher lung Th_17_ responses (150). In a mucosal DC vaccination model, BCGΔ*hip1* induced immune responses that significantly reduced Mtb burden following challenge (150). These studies suggest that impeding CD40-CD40L interactions allows Mtb to induce suboptimal immunity, and that adjuvants that augment CD40 during vaccination are likely to improve efficacy. Another cell surface protein implicated in dampening innate immunity is LprG, a lipoprotein that binds to TLR2 on macrophages and has been implicated in inhibiting MHC class II antigen presentation and phagosome/lysosome fusion (151–153). Deleting *lprG* in BCG led to higher levels of pro-inflammatory cytokines, lower bacterial burdens and higher Th_17_ responses following vaccination compared to BCG in murine models (154).

Many Mtb secreted proteins have also been implicated in evading host immune responses. SapM is secreted extracellularly *via* the SecA2 pathway and is involved in preventing phagosome maturation in macrophages (155, 156). Vaccination of mice with BCGΔ*sapM* increased protection following challenge and led to increased activation and recruitment of DCs (157, 158). Together, these studies illustrate that deleting immune evasion genes in the context of live attenuated vaccine strains can significantly improve efficacy and suggest that sequential deletion of multiple immune evasion genes in a single vaccine strain is likely to lead to synergistic effects that improve vaccine efficacy.

Studies focused on innate immune pathways modulated by Mtb may also provide key insights into strategies for enhancing innate immunity during vaccination. Adjuvants that trigger and engage specific DC responses during Mtb infection may be of particular interest as a way to combat Mtb induced delays in DC activation and suppression of antigen presentation to CD4^+^ T cells. Approaches to enhancing Th_17_ responses include adjuvants that engage CD40 on DCs (91) and MPL/chitosan formulations that induce Th_17_ polarising cytokines in DCs (114). Ligands that stimulate TLR7 and TLR9 on DCs have also been shown to upregulate MHCII and reduce anti-inflammatory IL-10 responses following BCG vaccination (159). Other receptors to target include inflammasome components and other cytosolic recognition receptors known to play a role in protective immunity to Mtb infection (160). Developing adjuvants that target additional immune evasion pathways in DCs, such as autophagy, can enhance antigen presentation (93). Targeting costimulatory and coinhibitory molecules on DCs has the potential to be beneficial in improving the immunogenicity of candidate vaccine by fine-tuning the pro- and anti-inflammatory pathways necessary for optimal immunity (91, 161–163). Additionally, adjuvants that limit early induction of IL-10 and T-regulatory cell expansion may be effective given the role of these responses in dampening immune responses during infection (164–166). Finally, we need a deeper understanding of the suppressive functions of neutrophils and MDSCs in order to develop strategies that target these cell types using adjuvant-like approaches or by targeted depletion.

In summary, studies on Mtb immune evasion mechanisms have given us valuable insights, not only on how Mtb manipulates host immunity, but have also identified pathogen and host targets for designing live attenuated vaccine strains and novel vaccine adjuvants that enhance vaccine efficacy.

## Animal Models

TB is a complex disease and no specific animal model perfectly mimics or recapitulates Mtb infection in humans. However, harnessing the strength of different animal models available will prove useful in developing and testing new adjuvants for TB.

NHPs have played a significant role in TB research and have been increasingly used for vaccine and adjuvant development. The strength of this NHP model lies in its ability to recapitulate disease similar to that in humans. NHPs can develop a latent version of Mtb infection, produce granulomatous lesions, and be used as a model to study HIV/TB co-infection. The use of primates has provided key insights such as the enhanced protective effect of intravenous BCG vaccination and the safety and efficacy of trial TB vaccines including M72/AS01_E_ and ID93 + GLA-SE (8, 167). Different routes of vaccination can be tested in NHPs, as the intravenous BCG study also included comparisons to intradermal, aerosol, and intradermal & aerosol routes of administration (8). NHPs have been used to test different vaccination strategies such as the prime-boost approach in which macaques are primed with BCG and then boosted with experimental vaccines and adjuvants 3-4 months after priming (168, 169). Additionally, NHP studies on protective vaccines can help inform immune correlates of protection, such as the study which observed protection using pulmonary-delivered BCG and the intravenous BCG study (8, 51). Given their similarity to humans, the NHP model is best equipped to study the priority areas outlined in this review. However, due to the prohibitive costs and challenges of working with primates (along with limited number of animals available for research), the use of small animal models should continue to be used to perform initial pre-clinical studies of potential adjuvants.

Historically, small animal models (mice and guinea pigs) have been used by developers to test adjuvant suitability for inducing protective immunity under rigorous *in vivo* conditions. The mouse model represents an excellent screen for first time *in vivo* assessment of a test vaccine. Using mice affords greater feasibility in testing of different variables such as the effect of different doses, alternative routes of inoculation and different inoculation times post-vaccination in a relatively short time frame. Additionally, the ability to use knockout and transgenic mice provides an additional level of interrogation for vaccine formulations and a better understanding of mechanisms involved in inducing protective immune responses (37). For instance, a recent analysis of the effect of intranasal vaccination using ODN as a molecular adjuvant showed that immunity could be achieved in a type I interferon-dependent manner (170). Another study demonstrated that intranasal vaccination was the best route for inducing protective immunity compared to intraperitoneal, subcutaneous, or intragastric routes (170). The cost of mouse models also allows for long-term immune profiling in vaccination studies. Extended timepoints have been previously been used to identify improved protective efficacy of subunit vaccine candidates compared to BCG (171). New approaches are also being used that allow mouse models to better mimic the heterogenous immune responses observed in humans. The diversity outbred (DO) mice, where initial breeding is done with inbred and wild-caught strains, have been developed with a level of genetic diversity similar to humans and NHPs. In a BCG vaccine study with DO mice, a diverse response was observed in which some vaccinated mice were protected and others were highly susceptible to Mtb challenge (172). Another attempt to improve the mouse model is the recent development of the ultra-low dose model. Infecting with 1-2 CFU of Mtb *via* aerosol, instead of the conventional ~100 CFU dose, causes mice to produce a heterogenous immune response and granulomatous structure in the lung similar to humans (173). The use of these and other mouse models will therefore continue to be important for testing novel adjuvants and vaccination strategies against TB.

Guinea pigs serve as an additional model and is traditionally employed after a vaccine formulation has demonstrated success in mice. Guinea pigs were originally used in TB research to understand disease pathogenesis due to their susceptibility to Mtb infection as well as their use in distinguishing “mammalian” bacilli from “avian type” bacilli due to their resistance to the latter (174). Subsequent studies by Smith and colleagues (175, 176) demonstrated that aerosol infection of guinea pigs with virulent Mtb resulted in pulmonary pathology similar to that observed in humans as well as a lethal course of disease (177). This model therefore allow researchers to test the ability of a vaccine formulation to limit disease, reduce Mtb burden, and prolong survival. The success of the guinea pig to identify vaccine candidates that have the potential to succeed in humans was demonstrated by a study which found the M72 vaccine candidate was able to boost BCG responses in the model (178). The long-term guinea pig model was used until recently to determine the efficacy of a vaccine as a low dose aerosol infection with virulent Mtb able to induce progressive pulmonary and extra-pulmonary disease similar to acute TB in humans (179). Vaccination with BCG resulted in the significant reduction of pathology and prolonged survival in guinea pigs, therefore validating the use of this model to test vaccines that can be efficacious in humans. Guinea pigs can therefore provide valuable insight into the ability of different formulations to significantly limit disease progression.

Together, the use of both mouse and guinea pig models of TB have contributed to a better understanding of adjuvant mechanisms in vaccines. The NIH/NIAID TB Vaccine testing program (180) has used both the C57BL/6 short-term mouse model and the Hartley guinea pig as a short- and long-term model to test vaccines and novel adjuvants. Both models have similar experimental strategies; animals are vaccinated with the test vaccine formulation, rested, and then infection is established by aerosol challenge with a low dose of Mtb (approximately 100 CFU for the mouse and 10 CFU for the guinea pig). The transition of testing adjuvants from the mouse to the guinea pig model has resulted in down selection of candidate vaccines under the NIH testing contracts and few have been able to reach the clinical testing stage. Of note has been the M72/AS01_E_ vaccine, which demonstrated activity in the mouse and guinea pig models (178). Using both models also reconcile differences observed experimentally. Recent investigations with ODN adjuvants have highlighted these discrepancies; when the same formulation and inoculation route were used in both models, the adjuvant proved less effective in guinea pigs. These results have been observed with other adjuvants and emphasize the need to test vaccines in both the mouse and guinea pig models While adjuvanted subunit vaccines have in general been less effective at limiting disease in the guinea pig model, it is not clear whether this is a limitation of the model or whether guinea pigs are selecting out formulations that may perform poorly in clinical trials. Unfortunately, there are only a small number of adjuvanted vaccines currently in clinical trials, some of which have been successfully tested in the guinea pig model (181, 182). An increased understanding of immune mechanisms in the guinea pig would be useful to determine if adjuvants were providing the appropriate signals to generate protective immunity. With the limited number of immunological tools available for the guinea pig to perform in-depth analyses (compared to mice), it is difficult to determine why such differences occur. Developing novel reagents for the guinea pig is an area that must be supported to achieve a better understanding of immunological responses to infection and vaccination. Additionally, a systematic analysis of cell phenotypes, expression of PRRs and other innate immune factors will be required to determine why adjuvants have provided limited protective immunity in the guinea pig model. This will help strengthen the use of this model for use in adjuvant studies for TB.

In summary, while NHP models more closely mimic human disease, small animal models provide feasability in testing vaccine adjuvants in a pre-clinical setting. With the rapid increase in the development of new molecular adjuvants, the mouse and guinea pig models provide the capacity to test them rigorously in an infectious disease setting. Harnessing the strength of all three of these animal models will therefore be crucial for developing new and improved adjuvants for TB vaccines.

## Conclusion

Recent advances in vaccine development, including an adjuvanted TB vaccine providing nearly 50% protection and observations on improving BCG, have reinvigorated the field. As our understanding of the complex interplay between Mtb and the host immune system is improved, new strategies for adjuvant development may be employed to further boost efficacy and develop a protective vaccine against TB.

In this review, we have highlighted four areas of importance for researchers in the TB field to address in order to generate more efficacious adjuvants and vaccination strategies. Defining correlates of protection is necessary to dictate which pathways should be targeted in order to induce protective immune responses. Similarly, learning how to target cells that are responsible for inducing those protective immune responses serves as a direct access to immune responses that are often dampened during infection. This is critical as Mtb is able to encode immune evasion proteins that serve to actively impede key immune cell subsets. Further characterizing these proteins give us direct insight into pathways that can be activated to overcome pathogen inhibition. Additionally, having appropriate animal models is necessary to test whether adjuvants and vaccines can be beneficial to humans while at the same time serving as a tool to further study infection and disease. While research into these topics can contribute directly to the generation of new adjuvants and vaccines, current adjuvants and vaccines are instructive in determining the requirements for protective immunity to TB. While intravenous delivery of BCG in humans is controversial, the study in NHPs (8) served as a model for identifying correlates of protection that can be targeted through safer, more practical means. Similarly, the M72/AS01_E_ vaccine study (10) demonstrated the benefit of adjuvants in TB vaccine strategies and contributed potential insights into mechanisms of protection. Cross-talk between basic TB science research and TB translational research should be encouraged as each of these inform the other. Additionally, insights from within the field are able to inform other fields that require generation of similar immune responses for their own models of protection. Therefore, advancing research in these four areas identified at the workshop has the potential to improve immunology in general while working to reduce TB burden worldwide.

## Author Contributions

ABE, AI, SMM, ELS, RNM, DJF, JTE, JR and JAT developed, wrote, and edited sections of the review as well as edited the entire manuscript. ABE provided significant revisions to the manuscript. All authors contributed to the article and approved the submitted version.

## Funding

The workshop and publication have been supported by the Division of AIDS, National Institute of Allergy and Infectious Diseases, National Institutes of Health, Department of Health and Human Services under the contract HHSN272201600001G.

## Conflict of Interest

Author RNM was employed by company Columbus Technologies & Services Inc. JE and SM are employees of and own shares in Inimmune Corporation.

The remaining authors declare that the research was conducted in the absence of any commercial or financial relationships that could be construed as a potential conflict of interest.

## Publisher’s Note

All claims expressed in this article are solely those of the authors and do not necessarily represent those of their affiliated organizations, or those of the publisher, the editors and the reviewers. Any product that may be evaluated in this article, or claim that may be made by its manufacturer, is not guaranteed or endorsed by the publisher.

## References

[1] Organization, W.H. Global Tuberculosis Report 2020. Geneva: WHO (2020).

[2] Pulendran B Ahmed R. Immunological Mechanisms of Vaccination. Nat Immunol (2011) 12(6):509–17. doi: 10.1038/ni.2039 PMC3253344 21739679

[3] Lu LL Chung AW Rosebrock TR Ghebremichael M YU WH Grace PS. A Functional Role for Antibodies in Tuberculosis. Cell (2016) 167(2):433–43.e14. doi: 10.1016/j.cell.2016.08.072 27667685 PMC5526202

[4] Urdahl KB Shafiani S Ernst JD. Initiation and Regulation of T-Cell Responses in Tuberculosis. Mucosal Immunol (2011) 4(3):288–93. doi: 10.1038/mi.2011.10 PMC3206635 21451503

[5] Griffiths KL Ahmed M Das S Gopal R Horne W Connell TD. Targeting Dendritic Cells to Accelerate T-Cell Activation Overcomes a Bottleneck in Tuberculosis Vaccine Efficacy. Nat Commun (2016) 7(1):13894. doi: 10.1038/ncomms13894 28004802 PMC5192216

[6] Jeyanathan M Yao Y Afkhami S Smaill F Xing Z. New Tuberculosis Vaccine Strategies: Taking Aim at Un-Natural Immunity. Trends Immunol (2018) 39(5):419–33. doi: 10.1016/j.it.2018.01.006 29429859

[7] Nemes E Geldenhuys H Rozot V Rutkowski KT Ratangee F Bilek N. Prevention of M. Tuberculosis Infection With H4:IC31 Vaccine or BCG Revaccination. N Engl J Med (2018) 379(2):138–49. 10.1056/NEJMoa1714021 PMC5937161 29996082

[8] Darrah PA Zeppa JJ Maiello P Hackney JA Wadsworth MH Hughes TK. Prevention of Tuberculosis in Macaques After Intravenous BCG Immunization. Nature (2020) 577(7788):95–102. doi: 10.1038/s41586-019-1817-8 31894150 PMC7015856

[9] Kaushal D Foreman TW Gautam US Alvarez X Adekambi T Rangel-Moreno J. Mucosal Vaccination With Attenuated Mycobacterium Tuberculosis Induces Strong Central Memory Responses and Protects Against Tuberculosis. Nat Commun (2015) 6(1):8533. doi: 10.1038/ncomms9533 26460802 PMC4608260

[10] Tait DR Hatherill M van der Meeren O Ginsberg AM Van Brakel E Salaun B. Final Analysis of a Trial of M72/AS01E Vaccine to Prevent Tuberculosis. N Engl J Med (2019) 381(25):2429–39. doi: 10.1056/NEJMoa1909953 31661198

[11] Didierlaurent AM Laupèze B Di Pasquale A Hergli N Collignon C Garçon N. Adjuvant System AS01: Helping to Overcome the Challenges of Modern Vaccines. Expert Rev Vaccines (2017) 16(1):55–63. doi: 10.1080/14760584.2016.1213632 27448771

[12] Luabeya AKK Kagina BMN Tameris MD Geldenhuys H Hoff ST Shi Z. First-In-Human Trial of the Post-Exposure Tuberculosis Vaccine H56:IC31 in Mycobacterium Tuberculosis Infected and non-Infected Healthy Adults. Vaccine (2015) 33(33):4130–40. doi: 10.1016/j.vaccine.2015.06.051 26095509

[13] Schellack C Prinz K Egyed A Fritz JH Wittmann B Ginzler M. IC31, a Novel Adjuvant Signaling via TLR9, Induces Potent Cellular and Humoral Immune Responses. Vaccine (2006) 24(26):5461–72. doi: 10.1016/j.vaccine.2006.03.071 16678312

[14] Aichinger MC Ginzler M Weghuber J Zimmermann L Riedl K Schütz G. Adjuvating the Adjuvant: Facilitated Delivery of an Immunomodulatory Oligonucleotide to TLR9 by a Cationic Antimicrobial Peptide in Dendritic Cells. Vaccine (2011) 29(3):426–36. doi: 10.1016/j.vaccine.2010.11.003 21093498

[15] Coccia M Collignon C Hervé C Chalon A Welsby I Detienne S. Cellular and Molecular Synergy in AS01-Adjuvanted Vaccines Results in an Early Ifnγ Response Promoting Vaccine Immunogenicity. NPJ Vaccines (2017) 2(1):25. doi: 10.1038/s41541-017-0027-3 29263880 PMC5627273

[16] Del Giudice G Rappuoli R Didierlaurent AM. Correlates of Adjuvanticity: A Review on Adjuvants in Licensed Vaccines. Semin Immunol (2018) 39:14–21. doi: 10.1016/j.smim.2018.05.001 29801750

[17] Macleod MKL McKee AS David A Wang J Mason R Kappler JW. Vaccine Adjuvants Aluminum and Monophosphoryl Lipid A Provide Distinct Signals to Generate Protective Cytotoxic Memory CD8 T Cells. Proc Natl Acad Sci (2011) 108(19):7914–9. doi: 10.1073/pnas.1104588108 PMC3093483 21518876

[18] Stewart E Triccas JA Petrovsky N. Adjuvant Strategies for More Effective Tuberculosis Vaccine Immunity. Microorganisms (2019) 7(8):255. doi: 10.3390/microorganisms7080255 PMC6724148 31409028

[19] Marty-Roix R Vladimer GI Pouliot K Weng D Buglione-Corbett R West K. Identification of QS-21 as an Inflammasome-Activating Molecular Component of Saponin Adjuvants. J Biol Chem (2016) 291(3):1123–36. doi: 10.1074/jbc.M115.683011 PMC4714196 26555265

[20] Didierlaurent AM Collignon C Bourguignon P Wouters S Fierens K Fochesato M. Enhancement of Adaptive Immunity by the Human Vaccine Adjuvant AS01 Depends on Activated Dendritic Cells. J Immunol (2014) 193(4):1920–30. doi: 10.4049/jimmunol.1400948 25024381

[21] Orr MT Desbien AL Cauwelaert ND Reed SG. Mechanisms of Activity of the Combination TLR4 Agonist and Emulsion Adjuvant GLA-SE. J Immunol (2016) 196(1 Supplement):75.2–2.

[22] Orr MT Duthie MS Windish HP Lucas EA Guderian JA Hudson TE. MyD88 and TRIF Synergistic Interaction is Required for TH1-Cell Polarization With a Synthetic TLR4 Agonist Adjuvant. Eur J Immunol (2013) 43(9):2398–408. doi: 10.1002/eji.201243124 PMC3803998 23716300

[23] Desbien AL Reed SJ Bailor HR Cauwelaert ND Laurance JD Orr MT. Squalene Emulsion Potentiates the Adjuvant Activity of the TLR4 Agonist, GLA, via Inflammatory Caspases, IL-18, and IFN-γ. Eur J Immunol (2015) 45(2):407–17. doi: 10.1002/eji.201444543 25367751

[24] Coler RN Day TA Ellis R Piazza FM Beckmann AM Vergara J. The TLR-4 Agonist Adjuvant, GLA-SE, Improves Magnitude and Quality of Immune Responses Elicited by the ID93 Tuberculosis Vaccine: First-in-Human Trial. NPJ Vaccines (2018) 3(1). doi: 10.1038/s41541-018-0057-5 PMC6123489 30210819

[25] Penn-Nicholson A Tameris M Smit E Day TA Musvosvi M Jayashankar L. Safety and Immunogenicity of the Novel Tuberculosis Vaccine ID93 + GLA-SE in BCG-Vaccinated Healthy Adults in South Africa: A Randomised, Double-Blind, Placebo-Controlled Phase 1 Trial. Lancet Respir Med (2018) 6(4):287–98. doi: 10.1016/S2213-2600(18)30077-8 29595510

[26] Perdomo C Zedler U Kühl AA Lozza L Saikali P Sander LE. Mucosal BCG Vaccination Induces Protective Lung-Resident Memory T Cell Populations Against Tuberculosis. mBio (2016) 7(6):e01686–16. doi: 10.1128/mBio.01686-16 PMC5120139 27879332

[27] Netea MG Joosten LAB Latz E Mills KHG Natoli G Stunnenberg HG. Trained Immunity: A Program of Innate Immune Memory in Health and Disease. Science (2016) 352(6284):aaf1098–aaf1098. doi: 10.1126/science.aaf1098 27102489 PMC5087274

[28] Kaufmann E Sanz J Dunn JL Khan N Mendonça LE Pacis A. BCG Educates Hematopoietic Stem Cells to Generate Protective Innate Immunity Against Tuberculosis. Cell (2018) 172(1-2):176–90.e19. doi: 10.1016/j.cell.2017.12.031 29328912

[29] Barclay WR Anacker RL Brehmer W Leif W Ribi E. Aerosol-Induced Tuberculosis in Subhuman Primates and the Course of the Disease After Intravenous BCG Vaccination. Infect Immun (1970) 2(5):574–82. doi: 10.1128/iai.2.5.574-582.1970 PMC416053 16557880

[30] Ribi E Anacker RL Barclay WR Brehmer W Harris SC Leif WR. Efficacy of Mycobacterial Cell Walls as a Vaccine Against Airborne Tuberculosis in the Rhesus Monkey. J Infect Dis (1971) 123(5):527–38. doi: 10.1093/infdis/123.5.527 5000470

[31] Anacker RL Brehmer W Barclay WR Leif WR Ribi E Simmons JH. Superiority of Intravenously Administered BCG and BCG Cell Walls in Protecting Rhesus Monkeys (Macaca Mulatta) Against Airborne Tuberculosis. Z Immunitatsforsch Exp Klin Immunol (1972) 143(4):363–76. 4282920

[32] Tameris MD Hatherill M Landry BS Scriba TJ Snowden MA Lockhart S. Safety and Efficacy of MVA85A, a New Tuberculosis Vaccine, in Infants Previously Vaccinated With BCG: A Randomised, Placebo-Controlled Phase 2b Trial. Lancet (2013) 381(9871):1021–8. doi: 10.1016/S0140-6736(13)60177-4 PMC5424647 23391465

[33] Christensen D Mortensen R Rosenkrands I Dietrich J Andersen P. Vaccine-Induced Th17 Cells are Established as Resident Memory Cells in the Lung and Promote Local IgA Responses. Mucosal Immunol (2017) 10(1):260–70. doi: 10.1038/mi.2016.28 27049058

[34] Lindenstrøm T Woodworth J Dietrich J Aagaard C Andersen P Agger EM. Vaccine-Induced Th17 Cells Are Maintained Long-Term Postvaccination as a Distinct and Phenotypically Stable Memory Subset. Infect Immun (2012) 80(10):3533–44. doi: 10.1128/IAI.00550-12 PMC3457559 22851756

[35] Van Dissel JT Joosten SA Hoff ST Soonawala D Prins C Hokey DA. A Novel Liposomal Adjuvant System, CAF01, Promotes Long-Lived Mycobacterium Tuberculosis-Specific T-Cell Responses in Human. Vaccine (2014) 32(52):7098–107. doi: 10.1016/j.vaccine.2014.10.036 25454872

[36] Pedersen GK Andersen P Christensen D. Immunocorrelates of CAF Family Adjuvants. Semin Immunol (2018) 39:4–13. doi: 10.1016/j.smim.2018.10.003 30396811

[37] Counoupas C Ferrell KC Ashhurst A Bhattacharyya ND Nagalingam G Stewart EL. Mucosal Delivery of a Multistage Subunit Vaccine Promotes Development of Lung-Resident Memory T Cells and Affords Interleukin-17-Dependent Protection Against Pulmonary Tuberculosis. NPJ Vaccines (2020) 5(1):105. doi: 10.1038/s41541-020-00255-7 33298977 PMC7665186

[38] Counoupas C Pinto R Nagalingam G Britton WJ Petrovsky N Triccas JA. Delta Inulin-Based Adjuvants Promote the Generation of Polyfunctional CD4+ T Cell Responses and Protection Against Mycobacterium Tuberculosis Infection. Sci Rep (2017) 7(1):8582. doi: 10.1038/s41598-017-09119-y 28819247 PMC5561132

[39] Tyne AS Chan JGY Shanahan ER Atmosukarto I Chan H-K Britton WJ. TLR2-Targeted Secreted Proteins From Mycobacterium Tuberculosis Are Protective as Powdered Pulmonary Vaccines. Vaccine (2013) 31(40):4322–9. doi: 10.1016/j.vaccine.2013.07.022 23880366

[40] Cooper AM. Cell-Mediated Immune Responses in Tuberculosis. Annu Rev Immunol (2009) 27(1):393–422. doi: 10.1146/annurev.immunol.021908.132703 19302046 PMC4298253

[41] Bloom B Zinkernagel R. Immunity to Infection. Curr Opin Immunol (1996) 8(4):465–6. doi: 10.1016/S0952-7915(96)80031-8 8794023

[42] Chackerian AA Perera TV Behar SM. Gamma Interferon-Producing CD4+ T Lymphocytes in the Lung Correlate With Resistance to Infection Withmycobacterium Tuberculosis. Infection Immun (2001) 69(4):2666–74. doi: 10.1128/IAI.69.4.2666-2674.2001 PMC98205 11254633

[43] Flynn JL Chan J Triebold KJ Dalton DK Stewart TA Bloom BR. An Essential Role for Interferon Gamma in Resistance to Mycobacterium Tuberculosis Infection. J Exp Med (1993) 178(6):2249–54. doi: 10.1084/jem.178.6.2249 PMC2191274 7504064

[44] Munk ME Emoto M. Functions of T-Cell Subsets and Cytokines in Mycobacterial Infections. Eur Respir J Suppl (1995) 20:668s–75s. 8590567

[45] Ottenhoff THM Kumararatne D Casanova J-L. Novel Human Immunodeficiencies Reveal the Essential Role of Type-1 Cytokines in Immunity to Intracellular Bacteria. Immunol Today (1998) 19(11):491–4. doi: 10.1016/S0167-5699(98)01321-8 9818540

[46] Counoupas C Triccas JA Britton WJ. Deciphering Protective Immunity Against Tuberculosis: Implications for Vaccine Development. Expert Rev Vaccines (2019) 18(4):353–64. doi: 10.1080/14760584.2019.1585246 30793629

[47] Andersen P Scriba TJ. Moving Tuberculosis Vaccines From Theory to Practice. Nat Rev Immunol (2019) 19(9):550–62. doi: 10.1038/s41577-019-0174-z 31114037

[48] Clemmensen HS Knudsen NPH Billeskov R Rosenkrands I Jungersen G Aagaard C. Rescuing ESAT-6 Specific CD4 T Cells From Terminal Differentiation Is Critical for Long-Term Control of Murine Mtb Infection. Front Immunol (2020) 11. doi: 10.3389/fimmu.2020.585359 PMC7677256 33240275

[49] Sallin MA Sakai S Kauffman KD Young HA Zhu J Barber DL. Th1 Differentiation Drives the Accumulation of Intravascular, Non-Protective CD4 T Cells During Tuberculosis. Cell Rep (2017) 18(13):3091–104. doi: 10.1016/j.celrep.2017.03.007 PMC5399512 28355562

[50] Gopal R Monin L Slight S Uche U Blanchard E Fallert Junecko BA. Unexpected Role for IL-17 in Protective Immunity Against Hypervirulent Mycobacterium Tuberculosis HN878 Infection. PloS Pathog (2014) 10(5):e1004099. doi: 10.1371/journal.ppat.1004099 24831696 PMC4022785

[51] Dijkman K Sombroek CC Vervenne RAW Hofman SO Boot C Remarque EJ. Prevention of Tuberculosis Infection and Disease by Local BCG in Repeatedly Exposed Rhesus Macaques. Nat Med (2019) 25(2). doi: 10.1038/s41591-018-0319-9 30664782

[52] Khader SA Guglani L Rangel-Moreno J Gopal R Junecko BA Fountain JJ. IL-23 and IL-17 in the Establishment of Protective Pulmonary CD4+ T Cell Responses After Vaccination and During Mycobacterium Tuberculosis Challenge. Nat Immunol (2007) 8(4):369–77. doi: 10.1038/ni1449 17351619

[53] Cooper AM Khader SA. The Role of Cytokines in the Initiation, Expansion, and Control of Cellular Immunity to Tuberculosis. Immunol Rev (2008) 226(1):191–204. doi: 10.1111/j.1600-065X.2008.00702.x 19161425 PMC4298252

[54] Arlehamn CL Seumois G Gerasimova A Huang C Fu Z Yue X. Transcriptional Profile of Tuberculosis Antigen–Specific T Cells Reveals Novel Multifunctional Features. J Immunol (2014) 193(6):2931–40. doi: 10.4049/jimmunol.1401151 PMC4157075 25092889

[55] Shanmugasundaram U Bucsan AN Ganatra SR Ibegbu C Quezada M Blair RV. Pulmonary Mycobacterium Tuberculosis Control Associates With CXCR3- and CCR6-Expressing Antigen-Specific Th1 and Th17 Cell Recruitment. JCI Insight (2020) 5(14). doi: 10.1172/jci.insight.137858 PMC7453885 32554933

[56] Cruz A Fraga AG Fountain JJ Rangel-Moreno J Torrado E Saraiva M. Pathological Role of Interleukin 17 in Mice Subjected to Repeated BCG Vaccination After Infection With Mycobacterium Tuberculosis. J Exp Med (2010) 207(8). doi: 10.1084/jem.20100265 PMC2916141 20624887

[57] Pollara G Turner CT Rosenheim J Chandran A Bell LCK Khan A. Exaggerated IL-17A Activity in Human *In Vivo* Recall Responses Discriminates Active Tuberculosis From Latent Infection and Cured Disease. Sci Trans Med (2021) 13(592). doi: 10.1126/scitranslmed.abg7673 PMC7610803 33952677

[58] Krensky AM Clayberger C. Biology and Clinical Relevance of Granulysin. Tissue Antigens (2009) 73(3):193–8. doi: 10.1111/j.1399-0039.2008.01218.x PMC2679253 19254247

[59] Chen CY Huang D Wang RC Shen L Zeng G Yao S. A Critical Role for CD8 T Cells in a Nonhuman Primate Model of Tuberculosis. PloS Pathog (2009) 5(4):e1000392. doi: 10.1371/journal.ppat.1000392 19381260 PMC2663842

[60] Lin PL Flynn JL. CD8 T Cells and Mycobacterium Tuberculosis Infection. Semin Immunopathol (2015) 37(3):239–49. doi: 10.1007/s00281-015-0490-8 PMC4439333 25917388

[61] Li H Javid B. Antibodies and Tuberculosis: Finally Coming of Age? Nat Rev Immunol (2018) 18(9):591–6. doi: 10.1038/s41577-018-0028-0 29872140

[62] Li H Wang X-X Wang B Fu L Liu G Lu Y. Latently and Uninfected Healthcare Workers Exposed to TB Make Protective Antibodies Against Mycobacterium Tuberculosis. Proc Natl Acad Sci (2017) 114(19):5023–8. doi: 10.1073/pnas.1611776114 PMC5441709 28438994

[63] Khader SA Guglani L Rangel-Moreno J Gopal R Junecko BA Fountain JJ. IL-23 Is Required for Long-Term Control of Mycobacterium Tuberculosis and B Cell Follicle Formation in the Infected Lung. J Immunol (2011) 187(10):5402–7. doi: 10.4049/jimmunol.1101377 PMC3208087 22003199

[64] Slight SR Rangel-Moreno J Gopal R Lin Y Fallert Junecko BA Mehra S. CXCR5+ T Helper Cells Mediate Protective Immunity Against Tuberculosis. J Clin Invest (2013) 123:712–6. doi: 10.1172/JCI65728 PMC3561804 23281399

[65] Rodo MJ Rozot V Nemes E Dintwe O Hatherill M Little F. A Comparison of Antigen-Specific T Cell Responses Induced by Six Novel Tuberculosis Vaccine Candidates. PloS Pathog (2019) 15(3):e1007643. doi: 10.1371/journal.ppat.1007643 30830940 PMC6417742

[66] Lo DD. Vigilance or Subversion? Constitutive and Inducible M Cells in Mucosal Tissues. Trends Immunol (2018) 39(3):185–95. doi: 10.1016/j.it.2017.09.002 28958392

[67] Nair VR Franco LH Zacharia VM Khan H Stamm CE Wu Y. Microfold Cells Actively Translocate Mycobacterium Tuberculosis to Initiate Infection. Cell Rep (2016) 16(5):1253–8. doi: 10.1016/j.celrep.2016.06.080 PMC4972672 27452467

[68] Zhai W Wu F Zhang Y Fu Y Liu Z. The Immune Escape Mechanisms of Mycobacterium Tuberculosis. Int J Mol Sci (2019) 20(2):340. doi: 10.3390/ijms20020340 PMC6359177 30650615

[69] Copland A. Mucosal Delivery of Fusion Proteins With Bacillus Subtilis Spores Enhances Protection Against Tuberculosis by Bacillus Calmette-Guérin. Front Immunol (2018) 9. doi: 10.3389/fimmu.2018.00346 PMC5857916 29593708

[70] Yang Q Zhang M Chen Q Chen W Wei C Qiao K. Cutting Edge: Characterization of Human Tissue-Resident Memory T Cells at Different Infection Sites in Patients With Tuberculosis. J Immunol (2020) 204(9):2331–6. doi: 10.4049/jimmunol.1901326 PMC7167459 32229539

[71] Ogongo P Tezera L Ardain A Nhamoyebonde S Ramsuran D Singh A. Tissue Resident-Like CD4+ T Cells Secreting IL-17 Control Mycobacteria Tuberculosis in the Human Lung. J Clin Invest (2021) 131:1–14. doi: 10.1172/JCI142014 PMC8121523 33848273

[72] Verrall AJ Netea G Alisjahbana B Hill PC Van Crevel R. Early Clearance Ofmycobacterium Tuberculosis: A New Frontier in Prevention. Immunology (2014) 141(4):506–13. doi: 10.1111/imm.12223 PMC3956425 24754048

[73] Khader SA Pearl JE Sakamoto K Gilmartin L Bell GK Jelley-Gibbs DM. IL-23 Compensates for the Absence of IL-12p70 and Is Essential for the IL-17 Response During Tuberculosis But Is Dispensable for Protection and Antigen-Specific IFN-γ Responses If IL-12p70 Is Available. J Immunol (2005) 175(2):788–95. doi: 10.4049/jimmunol.175.2.788 16002675

[74] Chackerian AA Chen S-J Brodie SJ Mattson JD Mcclanahan TK Kastelein RA. Neutralization or Absence of the Interleukin-23 Pathway Does Not Compromise Immunity to Mycobacterial Infection. Infection Immun (2006) 74(11):6092–9. doi: 10.1128/IAI.00621-06 PMC1695481 16923792

[75] Umemura M Yahagi A Hamada S Begum MD Watanabe H Kawakami K. IL-17-Mediated Regulation of Innate and Acquired Immune Response Against Pulmonary Mycobacterium Bovis Bacille Calmette-Guérin Infection. J Immunol (2007) 178(6):3786–96. doi: 10.4049/jimmunol.178.6.3786 17339477

[76] Happel KI Lockhart EA Mason CM Porretta E Keoshkerian E Odden AR. Pulmonary Interleukin-23 Gene Delivery Increases Local T-Cell Immunity and Controls Growth of Mycobacterium Tuberculosis in the Lungs. Inf Immun (2005) 73(9):5782–8. doi: 10.1128/IAI.73.9.5782-5788.2005 PMC1231058 16113296

[77] Van Den Berg RA. Adjuvant-Associated Peripheral Blood mRNA Profiles and Kinetics Induced by the Adjuvanted Recombinant Protein Candidate Tuberculosis Vaccine M72/AS01 in Bacillus Calmette–Guérin-Vaccinated Adults. Front Immunol (2018) 9. doi: 10.3389/fimmu.2018.00564 PMC5879450 29632533

[78] Billeskov R Lindenstrøm T Woodworth J Vilaplana C Cardona P-J Cassidy JP. High Antigen Dose Is Detrimental to Post-Exposure Vaccine Protection Against Tuberculosis. Front Immunol (2018) 8. doi: 10.3389/fimmu.2017.01973 PMC5775287 29379507

[79] Desel C Werninghaus K Ritter M Jozefowski K Wenzel J Russkamp N. The Mincle-Activating Adjuvant TDB Induces MyD88-Dependent Th1 and Th17 Responses Through IL-1r Signaling. PloS One (2013) 8(1):e53531. doi: 10.1371/journal.pone.0053531 23308247 PMC3538599

[80] Abraham S Juel HB Bang P Cheeseman HM Dohn RB Cole T. Safety and Immunogenicity of the Chlamydia Vaccine Candidate CTH522 Adjuvanted With CAF01 Liposomes or Aluminium Hydroxide: A First-in-Human, Randomised, Double-Blind, Placebo-Controlled, Phase 1 Trial. Lancet Infect Dis (2019) 19(10):1091–100. doi: 10.1016/S1473-3099(19)30279-8 31416692

[81] Schick J Schäfer J Alexander C Dichtl S Murray PJ Christensen D. Cutting Edge: TNF Is Essential for Mycobacteria-Induced MINCLE Expression, Macrophage Activation, and Th17 Adjuvanticity. J Immunol (2020) 205(2):323–8. doi: 10.4049/jimmunol.2000420 32540999

[82] Schoenen H Bodendorfer B Hitchens K Manzanero S Werninghaus K Nimmerjahn F. Cutting Edge: Mincle Is Essential for Recognition and Adjuvanticity of the Mycobacterial Cord Factor and its Synthetic Analog Trehalose-Dibehenate. J Immunol (2010) 184(6):2756–60. doi: 10.4049/jimmunol.0904013 PMC3442336 20164423

[83] Werninghaus K Babiak A Groß O Hölscher C Dietrich H Agger EM. Adjuvanticity of a Synthetic Cord Factor Analogue for Subunit Mycobacterium Tuberculosis Vaccination Requires Fcrγ–Syk–Card9–dependent Innate Immune Activation. J Exp Med (2009) 206(1):89–97. doi: 10.1084/jem.20081445 19139169 PMC2626670

[84] Smith AJ Miller SM Buhl C Child R Whitacre M Schoener R. Species-Specific Structural Requirements of Alpha-Branched Trehalose Diester Mincle Agonists. Front Immunol (2019) 10. doi: 10.3389/fimmu.2019.00338 PMC6403188 30873180

[85] Ryter KT Ettenger G Rasheed OK Buhl C Child R Miller SM. Aryl Trehalose Derivatives as Vaccine Adjuvants for Mycobacterium Tuberculosis. J Medicinal Chem (2020) 63(1):309–20. doi: 10.1021/acs.jmedchem.9b01598 PMC6952572 31809053

[86] Van Dis E Sogi KM Rae CS Sivick KE Surh NH Leong ML. STING-Activating Adjuvants Elicit a Th17 Immune Response and Protect Against Mycobacterium Tuberculosis Infection. Cell Rep (2018) 23(5):1435–47. doi: 10.1016/j.celrep.2018.04.003 PMC6003617 29719256

[87] Carroll EC Jin L Mori A Muñoz-Wolf N Oleszycka E Moran HBT. The Vaccine Adjuvant Chitosan Promotes Cellular Immunity via DNA Sensor cGAS-STING-Dependent Induction of Type I Interferons. Immunity (2016) 44(3):597–608. doi: 10.1016/j.immuni.2016.02.004 26944200 PMC4852885

[88] Mori A Oleszycka E Sharp FA Coleman M Ozasa Y Singh M. The Vaccine Adjuvant Alum Inhibits IL-12 by Promoting PI3 Kinase Signaling While Chitosan Does Not Inhibit IL-12 and Enhances Th1 and Th17 Responses. Eur J Immunol (2012) 42(10):2709–19. doi: 10.1002/eji.201242372 22777876

[89] de Martino M. Immune Response to Mycobacterium Tuberculosis: A Narrative Review. Front Pediatr (2019) 7(350). doi: 10.3389/fped.2019.00350 PMC6718705 31508399

[90] Manicassamy S Pulendran B. Modulation of Adaptive Immunity With Toll-Like Receptors. Semin Immunol (2009) 21(4):185–93. doi: 10.1016/j.smim.2009.05.005 PMC4125416 19502082

[91] Sia JK Bizzell E Madan-Lala R Rengarajan J. Engaging the CD40-CD40L Pathway Augments T-Helper Cell Responses and Improves Control of Mycobacterium Tuberculosis Infection. PloS Pathog (2017) 13(8):e1006530. doi: 10.1371/journal.ppat.1006530 28767735 PMC5540402

[92] Triccas JA Shklovskaya E Spratt J Ryan AA Palendira U Fazekas De Stgroth B. Effects of DNA- and Mycobacterium Bovis BCG-Based Delivery of the Flt3 Ligand on Protective Immunity to Mycobacterium Tuberculosis. Infection Immun (2007) 75(11):5368–75. doi: 10.1128/IAI.00322-07 PMC2168302 17724075

[93] Jagannath C Lindsey DR Dhandayuthapani S Xu Y Hunter RL Eissa NT. Autophagy Enhances the Efficacy of BCG Vaccine by Increasing Peptide Presentation in Mouse Dendritic Cells. Nat Med (2009) 15(3):267–76. doi: 10.1038/nm.1928 19252503

[94] Schreibelt G Tel J Sliepen K.H.E.W.J. Benitez-Ribas D Figdor CG Adema GJ. Toll-Like Receptor Expression and Function in Human Dendritic Cell Subsets: Implications for Dendritic Cell-Based Anti-Cancer Immunotherapy. Cancer Immunology Immunotherapy (2010) 59(10):1573–82. doi: 10.1007/s00262-010-0833-1 PMC11029854 20204387

[95] Sia JK Georgieva M Rengarajan J. Innate Immune Defenses in Human Tuberculosis: An Overview of the Interactions Between Mycobacterium Tuberculosis and Innate Immune Cells. J Immunol Res (2015) 2015:747543. doi: 10.1155/2015/747543 26258152 PMC4516846

[96] Domingo-Gonzalez R. Cytokines and Chemokines in Mycobacterium Tuberculosis Infection. Microbiol Spectr (2016) 4(5). doi: 10.1128/microbiolspec.TBTB2-0018-2016 PMC5205539 27763255

[97] Jakubzick CV Randolph GJ Henson PM. Monocyte Differentiation and Antigen-Presenting Functions. Nat Rev Immunol (2017) 17(6):349–62. doi: 10.1038/nri.2017.28 28436425

[98] Kadowaki N Ho S Antonenko S De Waal Malefyt R Kastelein RA Bazan F. Subsets of Human Dendritic Cell Precursors Express Different Toll-Like Receptors and Respond to Different Microbial Antigens. J Exp Med (2001) 194(6):863–70. doi: 10.1084/jem.194.6.863 PMC2195968 11561001

[99] Marakalala MJ Ndlovu H. Signaling C-Type Lectin Receptors in Antimycobacterial Immunity. PloS Pathog (2017) 13(6):e1006333. doi: 10.1371/journal.ppat.1006333 28640912 PMC5481012

[100] Kim S-H Jang Y-S. Antigen Targeting to M Cells for Enhancing the Efficacy of Mucosal Vaccines. Exp Mol Med (2014) 46(3):e85–5. doi: 10.1038/emm.2013.165 PMC3972786 24626171

[101] Bull NC Stylianou E Kaveh DA Pinpathomrat N Pasricha J Harrington-Kandt R. Enhanced Protection Conferred by Mucosal BCG Vaccination Associates With Presence of Antigen-Specific Lung Tissue-Resident PD-1+ KLRG1– CD4+ T Cells. Mucosal Immunol (2019) 12(2):555–64. doi: 10.1038/s41385-018-0109-1 PMC7051908 30446726

[102] Hart P Copland A Diogo GR Harris S Spallek R Oehlmann W. Nanoparticle-Fusion Protein Complexes Protect Against Mycobacterium Tuberculosis Infection. Mol Ther (2018) 26(3):822–33. doi: 10.1016/j.ymthe.2017.12.016 PMC5910664 29518353

[103] Lai R Afkhami S Haddadi S Jeyanathan M Xing Z. Mucosal Immunity and Novel Tuberculosis Vaccine Strategies: Route of Immunisation-Determined T-Cell Homing to Restricted Lung Mucosal Compartments. Eur Respir Rev (2015) 24(136):356–60. doi: 10.1183/16000617.00002515 PMC9487824 26028646

[104] Jeyanathan M Afkhami S Khera A Mandur T Damjanovic D Yao Y. CXCR3 Signaling Is Required for Restricted Homing of Parenteral Tuberculosis Vaccine–Induced T Cells to Both the Lung Parenchyma and Airway. J Immunol (2017) 199(7):2555–69. doi: 10.4049/jimmunol.1700382 28827285

[105] Haddadi S. Expression and Role of VLA-1 in Resident Memory CD8 T Cell Responses to Respiratory Mucosal Viral-Vectored Immunization Against Tuberculosis. Sci Rep (2017) 7(1). doi: 10.1038/s41598-017-09909-4 PMC5573413 28842633

[106] Haddadi S. Mucosal-Pull Induction of Lung-Resident Memory CD8 T Cells in Parenteral TB Vaccine-Primed Hosts Requires Cognate Antigens and CD4 T Cells. Front Immunol (2019) 10. doi: 10.3389/fimmu.2019.02075 PMC6747041 31552032

[107] Thakur A. Intrapulmonary (I.Pulmon.) Pull Immunization With the Tuberculosis Subunit Vaccine Candidate H56/CAF01 After Intramuscular (I.M.) Priming Elicits a Distinct Innate Myeloid Response and Activation of Antigen-Presenting Cells Than I.M. Or I.Pulmon. Prime Im. Front Immunol (2020) 11. doi: 10.3389/fimmu.2020.00803 PMC7221191 32457748

[108] Vierboom MPM. Evaluation of Heterologous Prime-Boost Vaccination Strategies Using Chimpanzee Adenovirus and Modified Vaccinia Virus for TB Subunit Vaccination in Rhesus Macaques. NPJ Vaccines (2020) 5(1). doi: 10.1038/s41541-020-0189-2 PMC7224290 32435513

[109] Kim S-H Jang Y-S. The Development of Mucosal Vaccines for Both Mucosal and Systemic Immune Induction and the Roles Played by Adjuvants. Clin Exp Vaccine Res (2017) 6(1):15. doi: 10.7774/cevr.2017.6.1.15 28168169 PMC5292352

[110] Lavelle EC Ward RW. Mucosal Vaccines — Fortifying the Frontiers. Nat Rev Immunol (2021), 1–15. doi: 10.1038/s41577-021-00599-8 34312520 PMC8312369

[111] Delaney S Biffen M Maltby J Bell J Asimus S Aggarwal A. Tolerability in Man Following Inhalation Dosing of the Selective TLR7 Agonist, AZD8848. BMJ Open Respir Res (2016) 3(1):e000113. doi: 10.1136/bmjresp-2015-000113 PMC4769423 26933507

[112] Leaker BR. Effects of the Toll-Like Receptor 7 (TLR7) Agonist, AZD8848, on Allergen-Induced Responses in Patients With Mild Asthma: A Double-Blind, Randomised, Parallel-Group Study. Respir Res (2019) 20(1). doi: 10.1186/s12931-019-1252-2 PMC6924002 31856838

[113] Woodrow KA Bennett KM Lo DD. Mucosal Vaccine Design and Delivery. Annu Rev BioMed Eng (2012) 14:17–46. doi: 10.1146/annurev-bioeng-071811-150054 22524387

[114] Ahmed M Jiao H Domingo-Gonzalez R Das S Griffiths KL Rangel-Moreno J. Rationalized Design of a Mucosal Vaccine Protects Against Mycobacterium Tuberculosis Challenge in Mice. J Leukocyte Biol (2017) 101(6):1373–81. doi: 10.1189/jlb.4A0616-270R PMC5433857 28258153

[115] Marasini N Kaminskas LM. Subunit-Based Mucosal Vaccine Delivery Systems for Pulmonary Delivery - Are They Feasible? Drug Dev Ind Pharm (2019) 45(6):882–94. doi: 10.1080/03639045.2019.1583758 30767591

[116] Reljic R Sibley L Huang J-M Pepponi I Hoppe A Hong HA. Mucosal Vaccination Against Tuberculosis Using Inert Bioparticles. Infection Immun (2013) 81(11):4071–80. doi: 10.1128/IAI.00786-13 PMC3811844 23959722

[117] Zygmunt BM Rharbaoui F Groebe L Guzman CA. Intranasal Immunization Promotes Th17 Immune Responses. J Immunol (2009) 183(11):6933–8. doi: 10.4049/jimmunol.0901144 19890060

[118] Maroof A Yorgensen YM Li Y Evans JT. Intranasal Vaccination Promotes Detrimental Th17-Mediated Immunity Against Influenza Infection. PloS Pathog (2014) 10(1):e1003875. doi: 10.1371/journal.ppat.1003875 24465206 PMC3900655

[119] Aguilo N Alvarez-Arguedas S Uranga S Marinova D Monzón M Badiola J. Pulmonary But Not Subcutaneous Delivery of BCG Vaccine Confers Protection to Tuberculosis-Susceptible Mice by an Interleukin 17–Dependent Mechanism. J Infect Dis (2016) 213(5):831–9. doi: 10.1093/infdis/jiv503 26494773

[120] Raeven RH Brummelman J Pennings JL van der Maas L Helm K Tilstra W. Molecular and Cellular Signatures Underlying Superior Immunity Against Bordetella Pertussis Upon Pulmonary Vaccination. Mucosal Immunol (2018) 11(3):979–93. doi: 10.1038/mi.2017.81 28930286

[121] Orr MT Beebe EA E. Hudson T Argilla D Huang P-WD Reese VA. Mucosal Delivery Switches the Response to an Adjuvanted Tuberculosis Vaccine From Systemic TH1 to Tissue-Resident TH17 Responses Without Impacting the Protective Efficacy. Vaccine (2015) 33(48):6570–8. doi: 10.1016/j.vaccine.2015.10.115 PMC4679420 26541135

[122] Macri C Dumont C Johnston AP Mintern JD. Targeting Dendritic Cells: A Promising Strategy to Improve Vaccine Effectiveness. Clin Trans Immunol (2016) 5(3):e66. doi: 10.1038/cti.2016.6 PMC4815026 27217957

[123] Boks MA Ambrosini M Bruijns SC Kalay H Van Bloois L Storm G. MPLA Incorporation Into DC-Targeting Glycoliposomes Favours Anti-Tumour T Cell Responses. J Controlled Release (2015) 216:37–46. doi: 10.1016/j.jconrel.2015.06.033 26151293

[124] Sehgal K Dhodapkar KM Dhodapkar MV. Targeting Human Dendritic Cells *In Situ* to Improve Vaccines. Immunol Lett (2014) 162(1):59–67. doi: 10.1016/j.imlet.2014.07.004 25072116 PMC4506641

[125] Stylianou E Pepponi I Van Dolleweerd CJ Paul MJ Ma JK Reljic R. Exploring the Vaccine Potential of Dec-205 Targeting in Mycobacterium Tuberculosis Infection in Mice. Vaccine (2011) 29(12):2279–86. doi: 10.1016/j.vaccine.2011.01.030 21272603

[126] Velasquez LN. Targeting Mycobacterium Tuberculosis Antigens to Dendritic Cells via the DC-Specific-ICAM3-Grabbing-Nonintegrin Receptor Induces Strong T-Helper 1 Immune Responses. Front Immunol (2018) 9. doi: 10.3389/fimmu.2018.00471 PMC5890140 29662482

[127] Ferluga J Yasmin H Al-Ahdal MN Bhakta S Kishore U. Natural and Trained Innate Immunity Against Mycobacterium Tuberculosis. Immunobiology (2020) 225(3):151951. doi: 10.1016/j.imbio.2020.151951 32423788

[128] Khader SA Divangahi M Hanekom W Hill PC Maeurer M Makar KW. Targeting Innate Immunity for Tuberculosis Vaccination. J Clin Invest (2019) 129(9):3482–91. doi: 10.1172/JCI128877 PMC6715374 31478909

[129] Mitroulis I Ruppova K Wang B Chen L-S Grzybek M Grinenko T. Modulation of Myelopoiesis Progenitors Is an Integral Component of Trained Immunity. Cell (2018) 172(1-2):147–61.e12. doi: 10.1016/j.cell.2017.11.034 29328910 PMC5766828

[130] Moorlag S.J.C.F.M. Khan N Novakovic B Kaufmann E Jansen T Van Crevel R. β-Glucan Induces Protective Trained Immunity Against Mycobacterium Tuberculosis Infection: A Key Role for IL-1. Cell Rep (2020) 31(7):107634. doi: 10.1016/j.celrep.2020.107634 32433977 PMC7242907

[131] Jeyanathan M Damjanovic D Shaler CR Lai R Wortzman M Yin C. Differentially Imprinted Innate Immunity by Mucosal Boost Vaccination Determines Antituberculosis Immune Protective Outcomes, Independent of T-Cell Immunity. Mucosal Immunol (2013) 6(3):612–25. doi: 10.1038/mi.2012.103 23131783

[132] Goldberg MF Saini NK Porcelli SA. Evasion of Innate and Adaptive Immunity by Mycobacterium Tuberculosis. Microbiol Spectr (2014) 2(5):1–24. doi: 10.1128/microbiolspec.MGM2-0005-2013 26104343

[133] Wolf AJ Linas B Trevejo-Nuñez GJ Kincaid E Tamura T Takatsu K. Mycobacterium Tuberculosis Infects Dendritic Cells With High Frequency and Impairs Their Function *In Vivo* . J Immunol (2007) 179(4):2509–19. doi: 10.4049/jimmunol.179.4.2509 17675513

[134] Bold TD Banaei N Wolf AJ Ernst JD. Suboptimal Activation of Antigen-Specific CD4+ Effector Cells Enables Persistence of M. Tuberculosis *In Vivo* . PloS Pathog (2011) 7(5):e1002063. doi: 10.1371/journal.ppat.1002063 21637811 PMC3102708

[135] Grace PS Ernst JD. Suboptimal Antigen Presentation Contributes to Virulence Ofmycobacterium tuberculosis *In Vivo* . J Immunol (2016) 196(1):357–64. doi: 10.4049/jimmunol.1501494 PMC4684992 26573837

[136] Madan-Lala R Sia JK King R Adekambi T Monin L Khader SA. Mycobacterium Tuberculosis Impairs Dendritic Cell Functions Through the Serine Hydrolase Hip1. J Immunol (2014) 192(9):4263–72. doi: 10.4049/jimmunol.1303185 PMC3995873 24659689

[137] Shen H Chen ZW. The Crucial Roles of Th17-Related Cytokines/Signal Pathways in M. Tuberculosis Infection. Cell Mol Immunol (2018) 15(3):216–25. doi: 10.1038/cmi.2017.128 PMC5843620 29176747

[138] Madan-Lala R Peixoto KV Re F Rengarajan J. Mycobacterium Tuberculosis Hip1 Dampens Macrophage Proinflammatory Responses by Limiting Toll-Like Receptor 2 Activation. Infection Immun (2011) 79(12):4828–38. doi: 10.1128/IAI.05574-11 PMC3232659 21947769

[139] Sia JK Rengarajan J. Immunology of Mycobacterium Tuberculosis Infections. Microbiol Spectr (2019) 7(4):1–37. doi: 10.1128/microbiolspec.GPP3-0022-2018 PMC6636855 31298204

[140] Upadhyay S Mittal E Philips JA. Tuberculosis and the Art of Macrophage Manipulation. Pathog Dis (2018) 76(4):1–12. doi: 10.1093/femspd/fty037 PMC6251593 29762680

[141] Magcwebeba T Dorhoi A Du Plessis N. The Emerging Role of Myeloid-Derived Suppressor Cells in Tuberculosis. Front Immunol (2019) 10:1–8. doi: 10.3389/fimmu.2019.00917 31114578 PMC6502992

[142] Lyadova IV. Neutrophils in Tuberculosis: Heterogeneity Shapes the Way? Mediators Inflamm (2017) 2017:1–11. doi: 10.1155/2017/8619307 PMC5463159 28626346

[143] Velmurugan K Chen B Miller JL Azogue S Gurses S Hsu T. Mycobacterium Tuberculosis nuoG Is a Virulence Gene That Inhibits Apoptosis of Infected Host Cells. PloS Pathog (2007) 3(7):e110. doi: 10.1371/journal.ppat.0030110 17658950 PMC1924871

[144] Miller JL Velmurugan K Cowan MJ Briken V. The Type I NADH Dehydrogenase of Mycobacterium Tuberculosis Counters Phagosomal NOX2 Activity to Inhibit TNF-α-Mediated Host Cell Apoptosis. PloS Pathog (2010) 6(4):e1000864. doi: 10.1371/journal.ppat.1000864 20421951 PMC2858756

[145] Blomgran R Desvignes L Broken V Ernst JD. Mycobacterium Tuberculosis Inhibits Neutrophil Apoptosis, Leading to Delayed Activation of Naive CD4 T Cells. Cell Host Microbe (2012) 11(1):81–90. doi: 10.1016/j.chom.2011.11.012 22264515 PMC3266554

[146] Gengenbacher M Nieuwenhuizen N Vogelzang A Liu H Kaiser P Schuerer S. Deletion of nuoG From the Vaccine Candidate Mycobacterium Bovis BCG Δ Urec:: Hly Improves Prot. mBio (2016) 7(3):e00679–16. doi: 10.1128/mBio.00679-16 PMC4895111 27222470

[147] Dutta NK Mehra S Martinez AN Alvarez X Renner NA Morici LA. The Stress-Response Factor SigH Modulates the Interaction Between Mycobacterium Tuberculosis and Host Phagocytes. PloS One (2012) 7(1):e28958. doi: 10.1371/journal.pone.0028958 22235255 PMC3250399

[148] Martinez AN Mehra S Kaushal D. Role of Interleukin 6 in Innate Immunity to Mycobacterium Tuberculosis Infection. J Infect Dis (2013) 207(8):1253–61. doi: 10.1093/infdis/jit037 PMC3693587 23359591

[149] Naffin-Olivos JL Georgieva M Goldfarb N Madan-Lala R Dong L Bizzell E. Mycobacterium Tuberculosis Hip1 Modulates Macrophage Responses Through Proteolysis of Groel2. PloS Pathog (2014) 10(5):e1004132. doi: 10.1371/journal.ppat.1004132 24830429 PMC4022732

[150] Bizzell E Sia JK Quezada M Enriquez A Georgieva M Rengarajan J. Deletion of BCG Hip1 Protease Enhances Dendritic Cell and CD4 T Cell Responses. J Leukocyte Biol (2018) 103(4):739–48. doi: 10.1002/JLB.4A0917-363RR PMC6457460 29345365

[151] Gehring AJ Dobos KM Belisle JT Harding CV Boom WH. Mycobacterium Tuberculosislprg (Rv1411c): A Novel TLR-2 Ligand That Inhibits Human Macrophage Class II MHC Antigen Processing. J Immunol (2004) 173(4):2660–8. doi: 10.4049/jimmunol.173.4.2660 15294983

[152] Gaur RL Ren K Blumenthal A Bhamidi S González-Nilo FD Jackson M. LprG-Mediated Surface Expression of Lipoarabinomannan Is Essential for Virulence of Mycobacterium Tuberculosis. PloS Pathog (2014) 10(9):e1004376. doi: 10.1371/journal.ppat.1004376 25232742 PMC4169494

[153] Shukla S Richardson ET Athman JJ Shi L Wearsch PA Mcdonald D. Mycobacterium Tuberculosis Lipoprotein LprG Binds Lipoarabinomannan and Determines Its Cell Envelope Localization to Control Phagolysosomal Fusion. PloS Pathog (2014) 10(10):e1004471. doi: 10.1371/journal.ppat.1004471 25356793 PMC4214796

[154] Martinot AJ Blass E Yu J Aid M Mahrokhian SH Cohen SB. Protective Efficacy of an Attenuated Mtb Δlprg Vaccine in Mice. PloS Pathog (2020) 16(12):e1009096. doi: 10.1371/journal.ppat.1009096 33315936 PMC7769599

[155] Vergne I Chua J Lee HH Lucas M Belisle J Deretic V. Mechanism of Phagolysosome Biogenesis Block by Viable Mycobacterium Tuberculosis. Proc Natl Acad Sci (2005) 102(11):4033–8. doi: 10.1073/pnas.0409716102 PMC554822 15753315

[156] Zulauf KE Sullivan JT Braunstein M. The SecA2 Pathway of Mycobacterium Tuberculosis Exports Effectors That Work in Concert to Arrest Phagosome and Autophagosome Maturation. PloS Pathog (2018) 14(4):e1007011. doi: 10.1371/journal.ppat.1007011 29709019 PMC5945054

[157] Festjens N Bogaert P Batni A Houthuys E Plets E Vanderschaeghe D. Disruption of the SapM Locus in Mycobacterium Bovis BCG Improves its Protective Efficacy as a Vaccine Against M. Tuberculosis. EMBO Mol Med (2011) 3(4):222–34. doi: 10.1002/emmm.201000125 PMC3377067 21328541

[158] Festjens N Vandewalle K Houthuys E Plets E Vanderschaeghe D Borgers K. SapM Mutation to Improve the BCG Vaccine: Genomic, Transcriptomic and Preclinical Safety Characterization. Vaccine (2019) 37(27):3539–51. doi: 10.1016/j.vaccine.2019.05.022 31122861

[159] Bakhru P Sirisaengtaksin N Soudani E Mukherjee S Khan A Jagannath C. BCG Vaccine Mediated Reduction in the MHC-II Expression of Macrophages and Dendritic Cells is Reversed by Activation of Toll-Like Receptors 7 and 9. Cell Immunol (2014) 287(1):53–61. doi: 10.1016/j.cellimm.2013.11.007 24384074 PMC4096037

[160] Burkert S Schumann RR. RNA Sensing of Mycobacterium Tuberculosis and Its Impact on TB Vaccination Strategies. Vaccines (2020) 8(1):67. doi: 10.3390/vaccines8010067 PMC7158685 32033104

[161] Jayaraman P Jacques MK Zhu C Steblenko KM Stowell BL Madi A. TIM3 Mediates T Cell Exhaustion During Mycobacterium Tuberculosis Infection. PloS Pathog (2016) 12(3):e1005490. doi: 10.1371/journal.ppat.1005490 26967901 PMC4788425

[162] Barber DL. Tuberculosis Following PD-1 Blockade for Cancer Immunotherapy. Sci Transl Med (2019) 11(475). doi: 10.1126/scitranslmed.aat2702 PMC7372940 30651320

[163] Kauffman KD. PD-1 Blockade Exacerbates Mycobacterium Tuberculosis Infection in Rhesus Macaques. Sci Immunol (2021) 6(55). doi: 10.1126/sciimmunol.abf3861 PMC8300572 33452107

[164] Redford PS Boonstra A Read S Pitt J Graham C Stavropoulos E. Enhanced Protection to Mycobacterium Tuberculosis Infection in IL-10-Deficient Mice is Accompanied by Early and Enhanced Th1 Responses in the Lung. Eur J Immunol (2010) 40(8):2200–10. doi: 10.1002/eji.201040433 PMC3378704 20518032

[165] Scott-Browne JP Shafiani S Tucker-Heard GS Ishida-Tsubota K Fontenot JD Rudensky AY. Expansion and Function of Foxp3-Expressing T Regulatory Cells During Tuberculosis. J Exp Med (2007) 204(9):2159–69. doi: 10.1084/jem.20062105 PMC2118702 17709423

[166] Shafiani S Tucker-Heard GS Kariyone A Takatsu K Urdahl KB. Pathogen-Specific Regulatory T Cells Delay the Arrival of Effector T Cells in the Lung During Early Tuberculosis. J Exp Med (2010) 207(7):1409–20. doi: 10.1084/jem.20091885 PMC2901066 20547826

[167] Foreman TW Mehra S Lackner AA Kaushal D. Translational Research in the Nonhuman Primate Model of Tuberculosis. ILAR J (2017) 58(2):151–9. doi: 10.1093/ilar/ilx015 PMC6279141 28575319

[168] Jeyanathan M Shao Z Yu X Harkness R Jiang R Li J. AdHu5Ag85A Respiratory Mucosal Boost Immunization Enhances Protection Against Pulmonary Tuberculosis in BCG-Primed Non-Human Primates. PloS One (2015) 10(8):e0135009. doi: 10.1371/journal.pone.0135009 26252520 PMC4529167

[169] Billeskov R Tan EV Cang M Abalos RM Burgos J Pedersen BV. Testing the H56 Vaccine Delivered in 4 Different Adjuvants as a BCG-Booster in a Non-Human Primate Model of Tuberculosis. PloS One (2016) 11(8):e0161217. doi: 10.1371/journal.pone.0161217 27525651 PMC4985151

[170] Troy A Esparza-Gonzalez SC Bartek A Creissen E Izzo L Izzo AA. Pulmonary Mucosal Immunity Mediated Through CpG Provides Adequate Protection Against Pulmonary Mycobacterium Tuberculosis Infection in the Mouse Model. A Role for Type I Interferon. Tuberculosis (2020) 123:101949. doi: 10.1016/j.tube.2020.101949 32741537 PMC7399211

[171] Counoupas C Pinto R Nagalingam G Hill-Cawthorne GA Feng CG Britton WJ. Mycobacterium Tuberculosis Components Expressed During Chronic Infection of the Lung Contribute to Long-Term Control of Pulmonary Tuberculosis in Mice. NPJ Vaccines (2016) 1(1):16012. doi: 10.1038/npjvaccines.2016.12 29263854 PMC5707878

[172] Kurtz SL Rossi AP Beamer GL Gatti DM Kramnik I Elkins KL. The Diversity Outbred Mouse Population Is an Improved Animal Model of Vaccination Against Tuberculosis That Reflects Heterogeneity of Protection. mSphere (2020) 5(2):e00097–20. doi: 10.1128/mSphere.00097-20 PMC7160682 32295871

[173] Plumlee CR Duffy FJ Gern BH Delahaye JL Cohen SB Stoltzfus CR. Ultra-Low Dose Aerosol Infection of Mice With Mycobacterium Tuberculosis More Closely Models Human Tuberculosis. Cell Host Microbe (2021) 29(1):68–82.e5. doi: 10.1016/j.chom.2020.10.003 33142108 PMC7854984

[174] Rich AR. The Pathogenesis of Tuberculosis. Oxford, UK: Oxford: Blackwell Scientific Publications (19511028). xxvii +.

[175] Smith DW Fok JS Ho RS Harding GE Wiegeshaus E Arora PK. Influence of BCG Vaccination on the Pathogenesis of Experimental Airborne Tuberculosis. J Hyg Epidemiol Microbiol Immunol (1975) 19(4):407–17. 812904

[176] Alsaadi AI Smith DW. The Fate of Virulent and Attenuated Mycobacteria in Guinea Pigs Infected by the Respiratory Route. Am Rev Respir Dis (1973) 107(6):1041–6. doi: 10.1164/arrd.1973.107.6.1041 4197232

[177] Harding GE Smith DW. Host-Parasite Relationships in Experimental Airborne Tuberculosis. VI. Influence of Vaccination With Bacille Calmette-Guerin on the Onset Andior Extent of Hematogenous Dissemination of Virulent Mycobacterium Tuberculosis to the Lungs. J Infect Dis (1977) 136(3):439–43. doi: 10.1093/infdis/136.3.439 409784

[178] Brandt L Alderson MR Lobet Y Dalemans W Turner OC. The Protective Effect of the Mycobacterium Bovis BCG Vaccine Is Increased by Coadministration With the Mycobacterium Tuberculosis 72-Kilodalton Fusion Polyprotein Mtb72F in M. Tuberculosis-Infected Guinea Pigs. Infection Immun (2004) 72(11):6622–32. doi: 10.1128/IAI.72.11.6622-6632.2004 PMC523007 15501795

[179] Smith DW Wiegeshaus E Navalkar R Grover AA. Host-Parasite Relationships in Experimental Airborne Tuberculosis. I. Preliminary Studies in BCG-Vaccinated and Nonvaccinated Animals. J Bacteriol (1966) 91(2):718–24. doi: 10.1128/jb.91.2.718-724.1966 PMC314919 4956758

[180] Izzo AA. Tuberculosis Vaccines — Perspectives From the NIH/NIAID Mycobacteria Vaccine Testing Program. Curr Opin Immunol (2017) 47:78–84. doi: 10.1016/j.coi.2017.07.008 28750280 PMC5626602

[181] Baldwin SL Bertholet S Reese VA Ching LK Reed SG Coler N. The Importance of Adjuvant Formulation in the Development of a Tuberculosis Vaccine. J Immunol (2012) 188(5):2189–97. doi: 10.4049/jimmunol.1102696 PMC3288309 22291184

[182] Vipond J Clark SO Hatch GJ Vipond R Marie Agger E Tree JA. Re-Formulation of Selected DNA Vaccine Candidates and Their Evaluation as Protein Vaccines Using a Guinea Pig Aerosol Infection Model of Tuberculosis. Tuberculosis (2006) 86(3-4):218–24. doi: 10.1016/j.tube.2006.01.014 16520093

